# Unravelling the connection between interferons and systemic lupus erythematosus: a systematic review and meta-analysis

**DOI:** 10.1186/s12916-025-04318-1

**Published:** 2025-10-08

**Authors:** Ridi Khatri, Anindita Banerjee, Naveen Khargekar, Shreyasi Athalye, Kirti Rai, Anjali Rajadhyaksha, Vijay Padwal, Vandana Pradhan

**Affiliations:** 1 Department of Clinical and Experimental Immunology, ICMR-National Institute of Immunohaematology, 13th Floor, New multi-storeyed building, KEM hospital campus, Mumbai, 400 012 India; 2 Department of Transfusion Transmitted Disease, ICMR-National Institute of Immunohaematology, Mumbai, India; 3 Department of Hematogenetics, ICMR-National Institute of Immunohaematology, Mumbai, India; 4 Department of Rheumatology, King Edward Memorial Hospital, Mumbai, India; 5 Library and IT, ICMR-National Institute of Immunohaematology, Mumbai, India

**Keywords:** Systemic lupus erythematosus, Interferons, Cytokines, Systematic review, Meta-analysis

## Abstract

**Background:**

Systemic lupus erythematosus (SLE) is characterized by dysregulated interferon (IFN) signaling. Despite its importance, a comprehensive and systematic synthesis of available data is lacking and findings across studies have been inconsistent. To address this gap, a systematic review and meta-analysis was conducted to evaluate global variations in IFNα, IFN-γ, and some important cytokines in adult SLE cases compared to healthy controls (HCs). Furthermore, we assessed their association with disease activity and effect of detection methods, sample types, and regional variations.

**Methods:**

A systematic search was conducted in PubMed and Scopus as primary databases, with Google Scholar used as a supplementary search engine, using MeSH terms and keywords related to SLE and IFNs (up to 15 November 2024). The Quantitative synthesis was performed using Comprehensive Meta-Analysis, calculating standardized mean differences (SMD) with 95% confidence intervals (CI) using a random-effects model for continuous outcomes. Correlation data were analyzed using Fisher’s *z* transformation. Publication bias was accessed using funnel plots and Egger’s test. For heterogeneity, Cochrane’s *Q* test, *I*^2^ statistic, subgroup analyses, sensitivity analyses, and Bayesian meta-analysis were conducted.

**Results:**

A total of 33 eligible studies, comparing IFN levels among 2307 SLE patients and 1599 HCs were included. Significantly elevated levels of IFNα (SMD = 1.428, 95%CI [0.78, 2.08], *p* < 0.001) and IFNγ (SMD = 0.922, 95%CI [0.32, 1.52], *p* = 0.003) in SLE patients compared with HCs were observed. Elevated levels of IFNγ were correlated with disease activity (SMD = 0.609, 95%CI [0.30, 0.91], *p* < 0.001). Additionally, significantly elevated levels of key pro-inflammatory cytokines, including IL-6 (SMD = 0.679, 95%CI [0.45, 0.90], *p* < 0.001) and TNFα (SMD = 1.754, 95%CI [0.25, 3.26], *p* = 0.022), were observed. Subgroup analyses revealed that differences in detection method, sample type, and geographic regions could influence measured cytokine levels.

**Conclusions:**

The elevated IFN levels in SLE patients, with a significant correlation of IFNγ with disease activity, suggest their role in disease pathogenesis and potential as a biomarker for monitoring disease activity. The findings identify IFNs and key pro-inflammatory cytokines as potential therapeutic targets. Given the limitations of our study, future research employing robust study designs and methodologies are warranted to increase the reliability of our findings.

**Trial registration:**

PROSPERO CRD42023445357.

**Supplementary Information:**

The online version contains supplementary material available at 10.1186/s12916-025-04318-1.

## Background

Systemic lupus erythematosus (SLE) is a complex autoimmune disease characterized by immune system dysregulation, leading to various clinical manifestations. It is marked by the generation of autoantibodies against various self-nuclear antigens and/or proteins and immune complex deposition, leading to inflammation [1]. Lupus is a multifactorial disease; however, exact mechanism of pathogenesis is not yet known [2–6]. Dysregulation of cytokine networks, particularly interferons (IFNs) and other inflammatory cytokines has also been reported in SLE pathogenesis [2, 4, 7–10]. To understand the underlying mechanism driving SLE pathogenesis and identify potential therapeutic targets, understanding the interplay between these cytokines and their association with disease activity is important.

IFNs are a group of signaling proteins crucial for mounting responses against viral infections and immune complexes, contributing to antiviral, inflammatory, and immunoregulatory functions [11]. They play an important role in connecting the responses of the adaptive and innate immune systems. Based on the receptor they bind, their unique structure, and biological functions, IFNs are classified into three types: type I (IFNα, IFNβ, IFNε, IFNκ, and IFNω), type II (IFNγ), and type III (IFN-λ1/IL-29, λ2/IL-28A, λ3/IL-28B, and λ4) [12]. In SLE, elevated levels of type I IFNs are frequently observed which is associated with increased disease activity, severity, and organ involvement [13–16]. Furthermore, an increase in the expression of IFN-regulated genes, commonly known as the “IFN signature,” is also observed in peripheral blood mononuclear cells (PBMCs), monocytes, and the blood of SLE patients [16–21], suggesting a significant role of the IFN pathway in immune dysregulation and the manifestation of diverse clinical outcomes. Moreover, the levels of type II and III IFNs are also observed to be elevated in SLE patients, which are associated with disease activity [11, 12, 15].

In addition to IFNs, SLE is characterized by the dysregulation of various other cytokines, mainly interleukin (IL)-1β, IL-6, IL-10, IL-12, IL-17, and tumor necrosis factor-alpha (TNFα) [4, 10]. Given their diverse roles in immune regulation, inflammation, tissue repair, and tolerance induction, their dysregulation in SLE leads to aberrant immune cell activation and tissue damage, exacerbating disease activity and progression [4, 9, 22]. Therefore, targeting these cytokines is promising for the management of SLE. The pro-inflammatory cytokine milieu plays a pivotal role in driving the pathogenesis of lupus, influencing disease activity, and serving as potential biomarker [4, 7, 22–26]. However, discrepancies in cytokine levels reported across studies and their inconsistent association with clinical parameters have limited their utility as reliable biomarkers. Several factors contribute to these discrepancies, including the study population, different detection methods, and the type of biological sample used [4].

The well-established role of IFNs in SLE pathogenesis is often obscured by inconsistent findings across individual studies and a lack of comprehensive and systematic synthesis of available data. In view of this, we conducted a systematic review and meta-analysis which aimed to understand the variations reported in different studies across the globe for IFNα, IFN-γ, and some important cytokines specifically in adult SLE cases compared to healthy controls (HCs). Additionally, we investigated the association of IFNs with disease activity and analyzed the effect of different detection methods, sample types, and regional variations on cytokine measurements.

## Methods

### Protocol and registration

This systematic review and meta-analysis was conducted in accordance with the Preferred Reporting Items for Systematic Reviews and Meta-Analyses (PRISMA) 2020 guidelines (see Additional file 1) [27]. The protocol used for the review was also registered with the International Prospective Register of Systematic Reviews, PROSPERO under registration number CRD42023445357.

### Strategy for retrieving information and criteria for selection

A comprehensive literature search was conducted in PubMed and Scopus as primary databases, with the last search performed on November 15, 2024. Additionally, Google Scholar was used as a supplementary search engine to identify any potentially missed relevant gray literature or articles cited in reference lists. The search strategy employed a combination of keywords and Medical Subject Headings (MeSH) terms, including: “Lupus erythematosus, systemic,” “Systemic Lupus Erythematosus,” “SLE,” “Lupus,” “Lupus erythematosus,” AND “Interferons” (see Additional file 2). We additionally reviewed the reference lists of relevant articles to ensure the thoroughness and comprehensiveness of the study.

After the initial search, the abstracts of all the results from each database were retrieved and uploaded to the web version of Rayyan software (https://rayyan.ai/) [28]. The initial screening of articles included the removal of duplicates, followed by an abstract review and sorting based on the inclusion and exclusion criteria. This activity was independently performed by two reviewers. In cases of conflict regarding the inclusion of the article, the opinion of a third reviewer was taken into consideration. Studies that met the following inclusion criteria were considered:

i. Participants aged ≥18 years;
ii. Study subjects: humans;
iii. Studies that have measured either type of IFN;
iv. Sample: serum or plasma;
v. Study Type: Cross-sectional, prospective, case-control, or cohort studies;
vi. Data: Serum or plasma levels of IFNs reported as mean and SD in SLE cases and HCs;

The following exclusion criteria were used for excluding any abstract from this systematic review and meta-analysis:

i. Pregnant, pediatric, or overlapping SLE patients;
ii. Clinical trials, case reports, reviews, letters/comments, conference abstracts;
iii. Studies without HCs or reporting zero mean IFN levels in HCs
iv. Insufficient data for meta-analysis (absence of mean and SD reporting for serum IFN levels)

### Extraction and evaluation of data quality

The full texts of the eligible abstracts were retrieved for evaluation and final inclusion in this systematic review and meta-analysis. Two reviewers independently extracted and cross-verified the data. The data were extracted in a spreadsheet where the following information was recorded: first author, year of publication, country, study design, number of SLE cases and HCs, sample type, mean and SD of IFNs and other cytokine levels (pg/mL), method used for IFNs detection and the Pearson/Spearman correlation coefficient between IFN and disease activity. In cases of conflict, consensus was achieved through detailed discussion between the reviewers and an independent review by a third reviewer.

The quality and risk of bias of the included studies were assessed using the Newcastle–Ottawa Quality Assessment Scale (NOS) [29]. Studies with scores equal to or above the median NOS value were categorized as high quality, while those scoring below the median were classified as low quality.

### Statistical analysis

The quantitative synthesis of data was performed using the Comprehensive Meta-Analysis V4 software. The standardized mean difference (SMD) with a 95% confidence interval (CI) was used to summarize the continuous outcomes in the data or assess the differences in IFNs and cytokines levels between groups, using the random effects model. The effect size was calculated using the inverse variance with Hedges’g correction method. To analyze the correlation between IFN levels and disease activity, the inverse variance method with Fisher’s *z* transformation was used. To assess the publication bias, funnel plots and Egger’s regression test were used [30]. To account for the asymmetry in funnel plots, trim-and-fill method was used [31].

Cochrane’s *Q* test, the *I*^2^ statistic, subgroup analyses (based on detection methods, sample type, and study regions), sensitivity analyses, and Bayesian meta-analyses were conducted to explore the sources of heterogeneity. The percentage of variation across studies was defined as low, moderate, or high based on *I*^2^ statistic values of 25, 50, and 75%, respectively. Sensitivity analysis was performed to assess the influence of each study on the pooled results. Given the high between-study heterogeneity observed in our analyses (*I*^2^ > 90% for all cytokines), we employed Bayesian hierarchical models with weakly informative priors to stabilize parameter estimation while maintaining model flexibility. This approach is well-supported in meta-analytic literature as a means to address heterogeneity without overly constraining the data [32, 33]. The analyses were conducted in R using the metafor, dplyr, and ggplot2 packages for classical meta-analysis and data visualization, and the brms package for Bayesian modeling. Models were fit using Hamiltonian Monte Carlo with four chains and 8000 post-warmup draws via the Stan backend. Posterior estimates of between-study variance (τ^2^) and pooled effect sizes demonstrated good convergence (R̂ ≈ 1.00), and full 95% credible intervals are reported to reflect parameter uncertainty. A *p*-value < 0.05 was considered as statistically significant.

## Results

### Studies screened and included in the meta-analysis

**Fig. 1:**
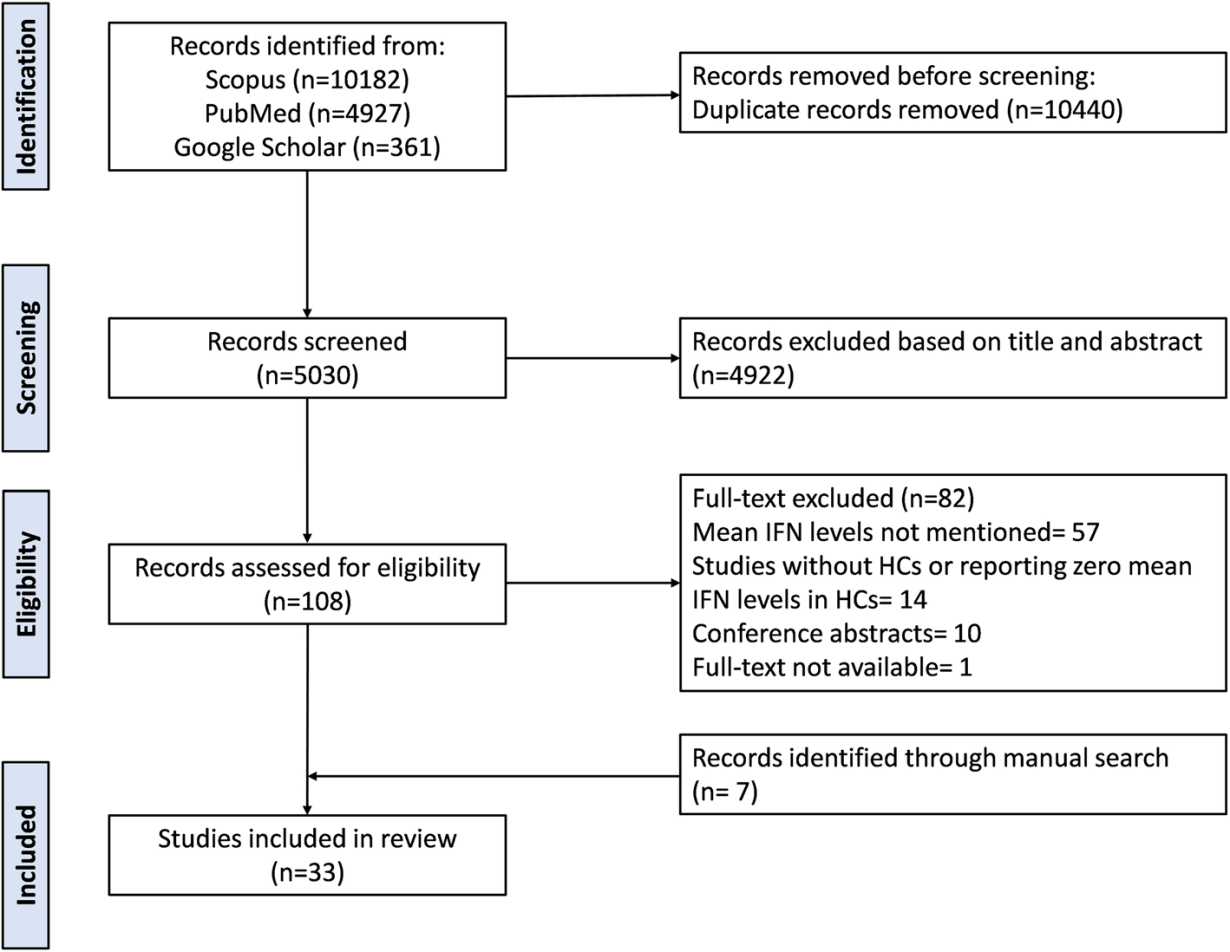
PRISMA 2020 flow diagram of the study selection process

The initial search strategy identified a total of 15,470 potentially relevant studies. Next, 10,440 studies were removed as duplicates. Based on the titles and abstracts, 4922 studies were excluded because of their lack of relevance to our research topic. A full-text examination of 108 studies was subsequently performed for eligibility, of which 82 studies were excluded (see Additional file 3) for the following reasons: 57 studies did not mention mean IFN levels; 14 studies either lacked healthy control groups or reported that the mean IFN levels in HCs were zero; ten studies were conference abstracts and one study had no full-text available. Through manual searches, seven additional articles were identified, leading to a final inclusion of 33 articles in the present systematic review and meta-analysis. The study selection process and inclusion of relevant studies are detailed in The PRISMA 2020 flow diagram (Fig. 1).

### Characteristics and quality assessment of the included studies

The characteristics of the studies included in this meta-analysis are summarized in additional file 4. In this systematic review and meta-analysis, we incorporated 33 studies comprising 19 cross-sectional, 12 case–control, and two cohort studies, published between 2002 and 2023. Collectively, these studies assessed and compared the mean levels of IFNs in 2307 SLE cases and 1599 HCs. The studies primarily assessed IFN levels in serum and plasma samples. Importantly, all the studies reported the mean ± SD levels of IFNs and other cytokines. The most commonly employed method to assess IFN levels was ELISA (*N* = 28, 84.8%), followed by bead-based assay (*N* = 5, 15.2%). Of the included studies, 12 were from Asia [34–45], nine from the Middle East and North America (MENA) [46–54], seven from America [55–61], and five from Europe [62–66].

The quality of included studies was evaluated using the NOS and the median NOS score was calculated to be 5. Based on this evaluation, 31 studies with a score of 5 or above were classified as high quality, indicating a low risk of bias. Meanwhile, two studies scored below 5 and were classified as poor quality, reflecting a high risk of bias (see Additional file 5). Additional file 6 presents the summary statistics of the meta-analysis.

### IFN levels in SLE cases compared with HCs and their correlation with disease activity:

**Fig. 2:**
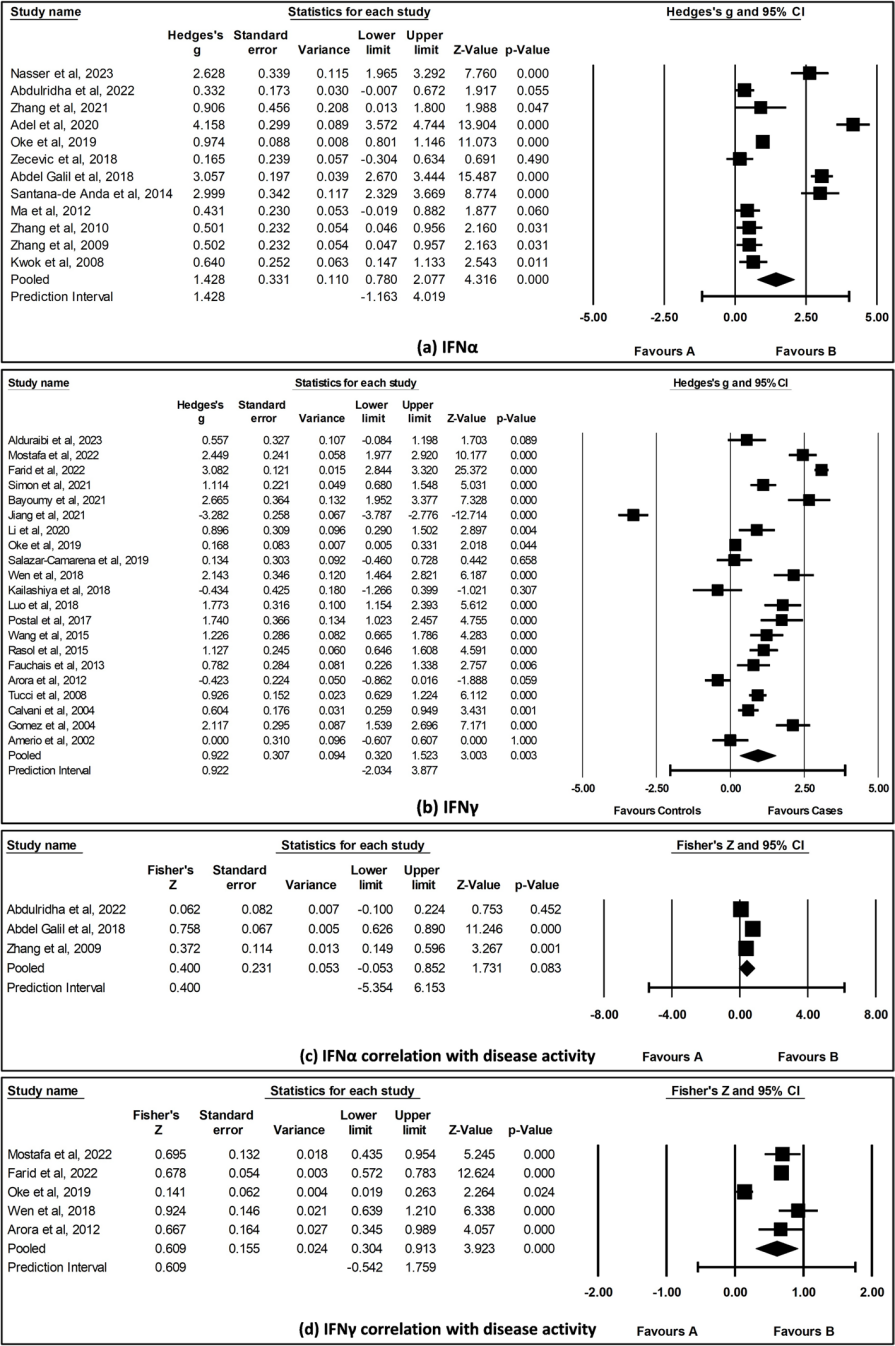
Forest plot of **a** IFNα and **b** IFNγ levels between SLE cases and HCs. Forest plot showing correlation of **c** IFNα and **d** IFNγ levels with disease activity. The pooled effect size was estimated via random-effects model and correlation was analyzed with inverse variance method

A meta-analysis of a total of 12 studies comparing IFNα levels and 21 studies comparing IFNγ levels between SLE cases and HCs was conducted. As shown in Fig. 2 a and b, the computed SMD and 95% CI for each of these studies indicated that there was a significant increase in IFNα levels (SMD = 1.428, 95% CI [0.78, 2.08], *I*^2^ = 96.5%, *p* < 0.001) and IFNγ levels (SMD = 0.922, 95% CI [0.32, 1.52], *I*^2^ = 97.6%, *p* = 0.003) in SLE cases compared with HCs.

Further, the meta-analysis included a total of three studies for IFNα and five studies for IFNγ levels assessing the association with disease activity (Fig. 2c, d). The results revealed a significant association between IFNγ levels and disease activity (SMD = 0.609, 95% CI [0.30, 0.91], *I*^2^ = 92.8%, *p* < 0.001).

### Cytokine levels in SLE cases compared with HCs

**Fig. 3:**
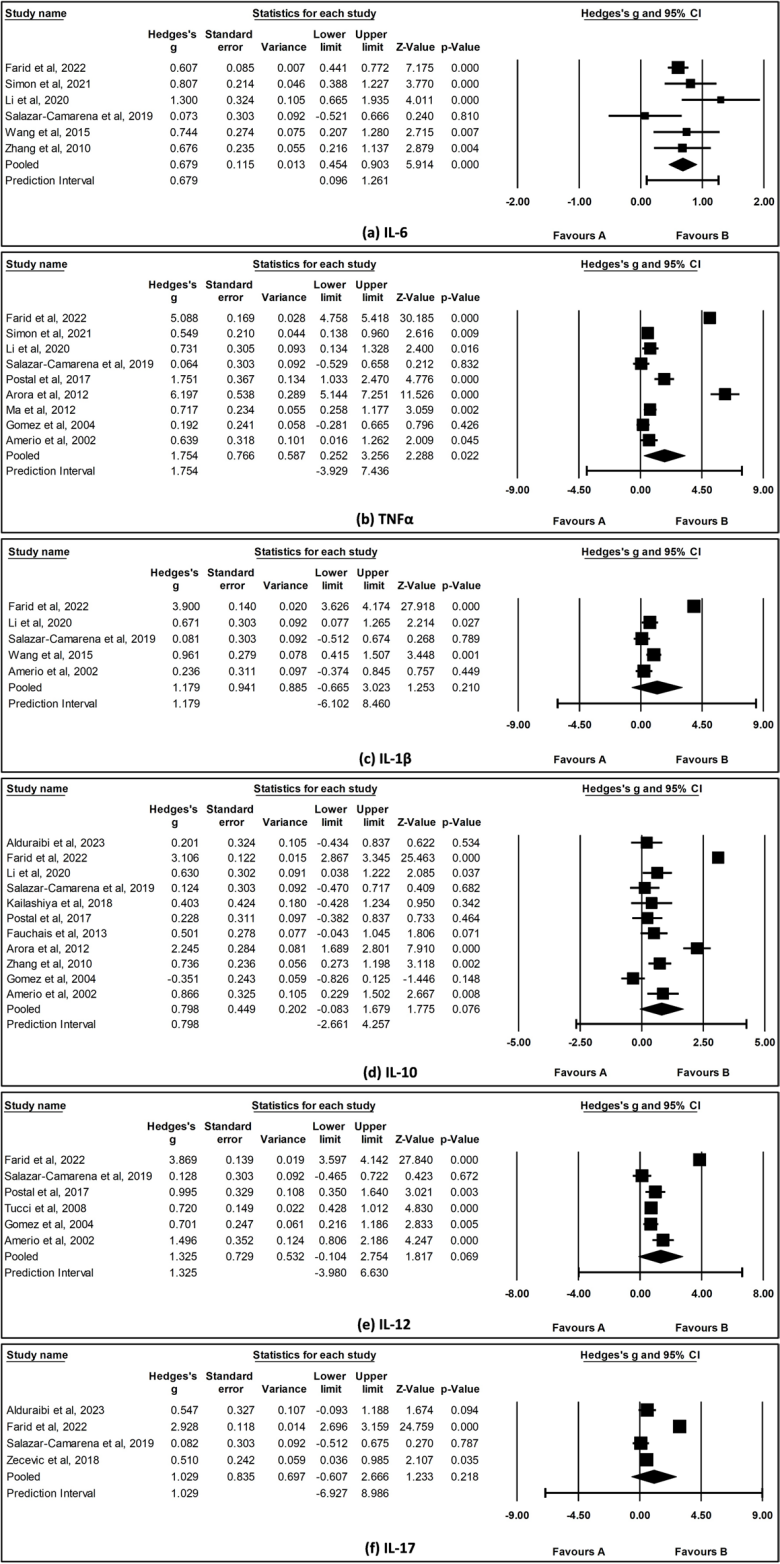
Forest plot of **a** IL-6, **b** TNFα, **c** IL-1β, **d** IL-10, **e** IL-12, and **f** IL-17 levels between SLE cases and HCs. The pooled effect size was estimated via random-effects model

As illustrated in Fig. 3 a and b, IL-6 (SMD = 0.679, 95% CI [0.45, 0.90], *I*^2^ = 41.8%, *p* < 0.001) and TNFα (SMD = 1.754, 95% CI [0.25, 3.26], *I*^2^ = 98.7%, *p* = 0.022) levels were significantly elevated in SLE cases compared with HCs. In contrast, IL-1β (SMD = 1.179, 95% CI [*− *0.67, 3.02], *I*^2^ = 98.6%, *p* = 0.210), IL-10 (SMD = 1.798, 95% CI [− 0.08, 1.68], *I*^2^ = 97.0%, *p* = 0.076), IL-12 (SMD = 1.325, 95% CI [− 0.10, 2.75], *I*^2^ = 98.5%, *p* = 0.069), and IL-17 (SMD = 1.029, 95% CI [− 0.61, 2.67], *I*^2^ = 98.1%, *p* = 0.218) levels did not show a significant increase in SLE cases (Fig. 3c–f).

### Sub-group analysis of cytokines by detection methods

**Fig. 4:**
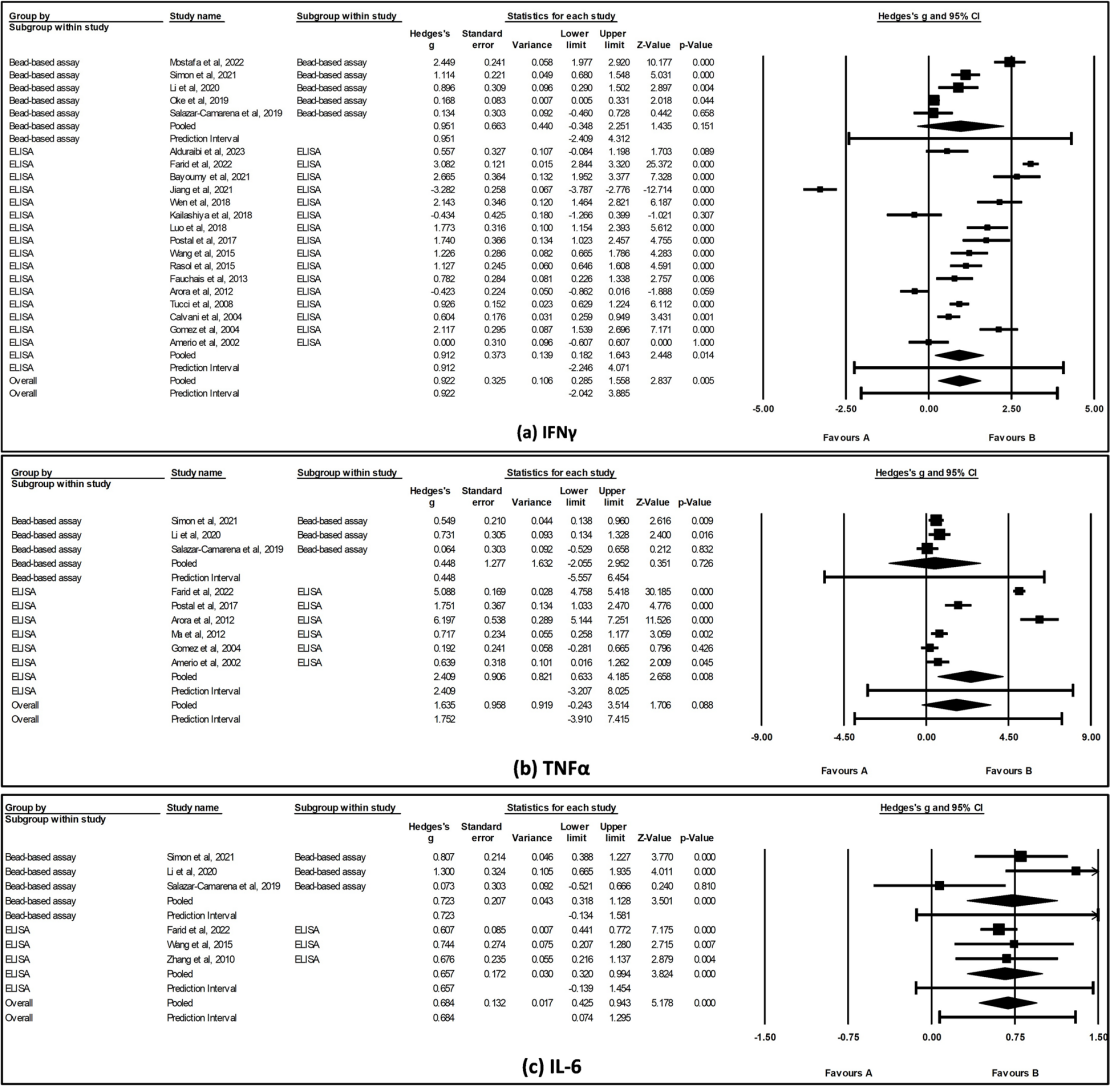
Forest plot showing subgroup analysis of **a** IFNγ, **b** TNFα, and **c** IL-6 levels by detection method. The pooled effect size was estimated via random-effects model

To investigate the potential sources of heterogeneity for the overall pooled results, we conducted sub-group analyses based on the detection method used for IFNγ, IL-6, and TNFα. The results showed a significant difference when ELISA was used for the detection of IFNγ (SMD = 0.912, 95% CI [0.18, 1.64], *p* = 0.014) and TNFα (SMD = 2.409, 95% CI [0.63, 4.19], *p* = 0.008). However, no significant difference was observed with bead-based assay for IFNγ (SMD = 0.951, 95% CI [− 0.35, 2.25], *p* = 0.151) and TNFα (SMD = 0.448, 95% CI [− 2.06, 2.95], *p* = 0.726) (Fig. 4a and b). Further, we observed that significant heterogeneity still existed for IFNγ regardless of the detection method used (*p* < 0.001), suggesting detection method may not fully account for the observed variability. However, for TNFα, heterogeneity was not significant when bead-based assay was used (*p* = 0.263), suggesting that the detection method could contribute to the variability observed (see Additional file 6). The findings for IL-6 were consistent with the overall effects; a significant difference was noted when either ELISA (SMD = 0.657, 95% CI [0.32, 0.99], *p* < 0.001) or bead-based assay (SMD = 0.723, 95% CI [0.32, 1.13], *p* < 0.001) was used (Fig. 4 c). Notably, when IL-6 levels were assessed using ELISA, no significant heterogeneity was observed (*p* = 0.868), suggesting that the detection method may influence the variability across studies.

### Sub-group analysis of cytokines by sample type

**Fig. 5:**
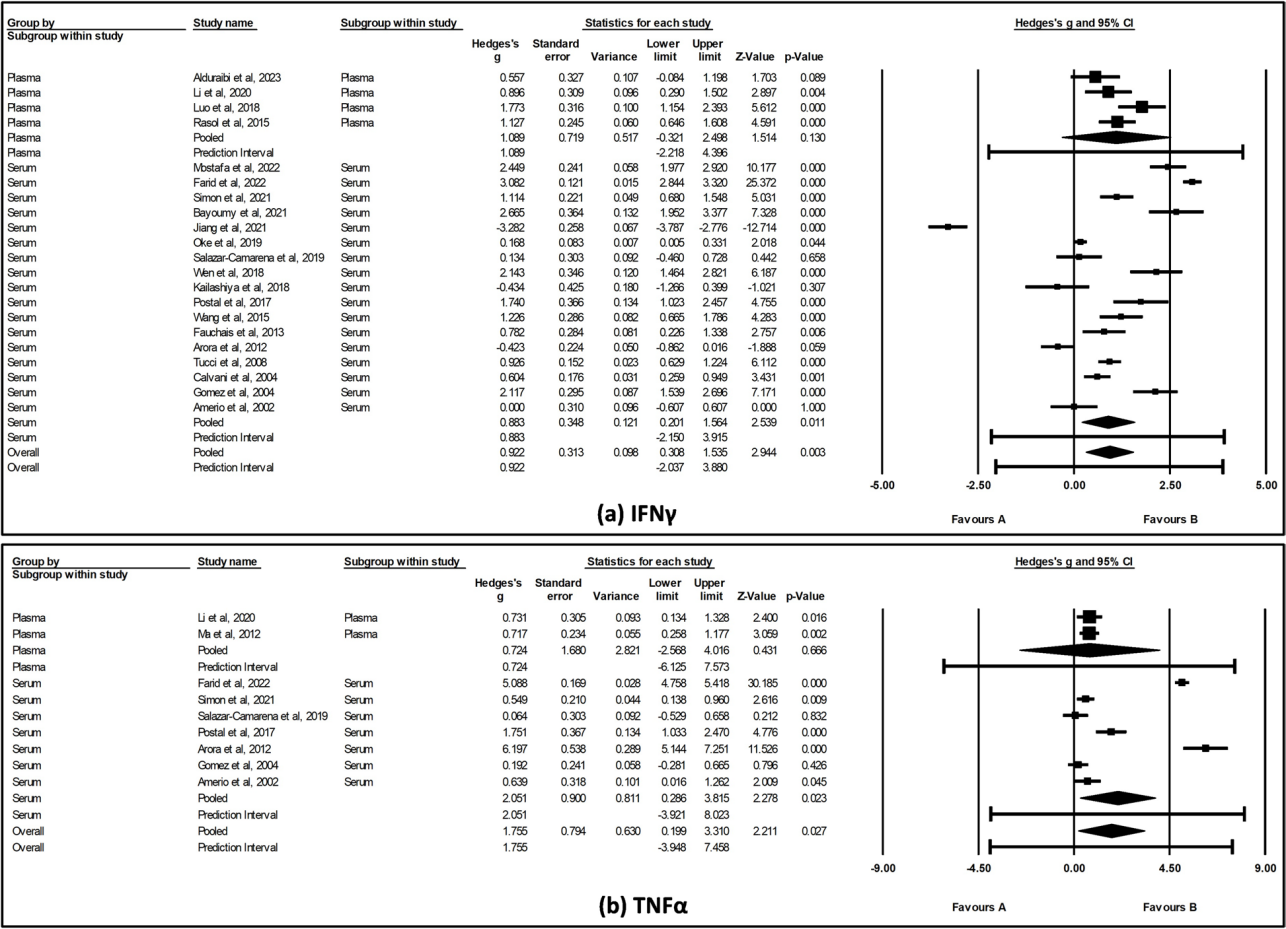
Forest plot showing subgroup analysis of **a** IFNγ and **b** TNFα levels by sample type. The pooled effect size was estimated via random-effects model

To further investigate the potential sources of heterogeneity, we conducted sub-group analyses based on the sample type used for assessing IFNγ and TNFα levels. The results showed significant differences for both IFNγ (SMD = 0.883, 95% CI [0.20, 1.56], *p* = 0.011) and TNFα (SMD = 2.051, 95% CI [0.29, 3.82], *p* = 0.023) when serum samples were used (Fig. 5a, b). In contrast, no significant differences were observed with plasma samples. Notably, heterogeneity was not significant when IFNγ and TNFα levels were assessed in plasma (*p* = 0.051 and *p* = 0.972, respectively), suggesting that the sample type may contribute to the observed variability (see Additional file 6).

### Sub-group analysis of cytokines by regional variations

**Fig. 6:**
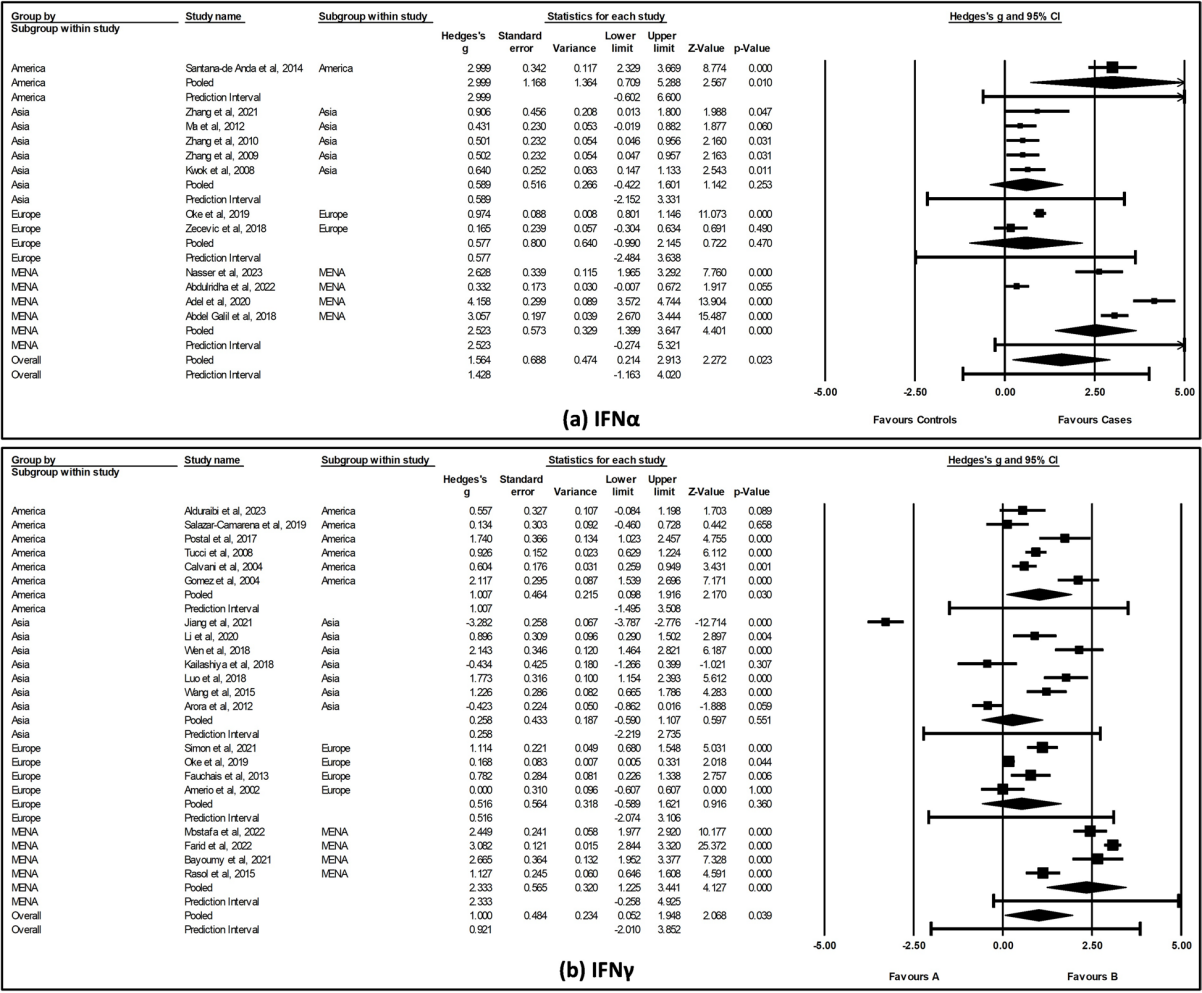
Forest plot showing subgroup analysis of **a** IFNα and **b** IFNγ levels across various populations. The pooled effect size was estimated via random-effects model

To assess whether regional differences contribute to heterogeneity, sub-group analyses were conducted for IFNα and IFNγ. Elevated IFNα levels were observed in SLE cases across various populations, with a statistically significant increase in American (SMD = 2.999, 95% CI [0.71, 5.29], *p* = 0.010) and MENA (SMD = 2.523, 95% CI [1.39, 3.65], *p* < 0.001) populations (Fig. 6a). Similarly, the regional sub-group analysis revealed significantly elevated IFNγ levels in both American (SMD = 1.007, 95% CI [0.09, 1.92], *p* = 0.030) and MENA (SMD = 2.333, 95% CI [1.23, 3.44], *p* < 0.001) populations (Fig. 6b). Notably, when IFNα levels were assessed in American and Asian populations, no significant heterogeneity was observed (*p* = 1.000 and *p* = 0.897, respectively), suggesting that the regional variation may account for the observed variability. In contrast, significant heterogeneity persisted for IFNγ across all regional sub-groups (*p* < 0.001), suggesting regional variations may not fully explain the observed heterogeneity (see Additional file 6).

### Bayesian sensitivity analysis

To address limitations of frequentist models under high heterogeneity, we conducted Bayesian meta-analyses using hierarchical models with weakly informative priors for IFN-α, IFN-γ, IL-6, and TNF-α. As shown in Table 1, posterior mean estimates were broadly consistent with frequentist random-effects estimates. For IFN-α, IFN-γ, and IL-6, the posterior distributions excluded zero, providing strong evidence for elevated cytokine levels in cases relative to controls. In contrast, the posterior estimate for TNF-α was more uncertain, with a 95% credible interval crossing zero—suggesting either weaker effects or greater underlying variability, possibly due to fewer included studies and methodological heterogeneity.

**Table 1:** Comparison of random-effects and Bayesian meta-analysis estimates for IFN-α, IFN-γ, IL-6, and TNF-α

Cytokine	Random-effects estimate (95% CI)	Bayesian posterior mean (95% CI)
IFN-α	1.428 [0.78, 2.08]	1.20 [0.40, 1.93] (SE = 0.39)
IFN-γ	0.922 [0.32, 1.52]	0.86 [0.25, 1.45] (SE = 0.31)
IL-6	0.679 [0.45, 0.90]	0.66 [0.36, 0.97] (SE = 0.15)
TNF-α	1.754 [0.25, 3.26]	1.10 [–0.26, 2.31] (SE = 0.65)

### Sensitivity analysis and publication bias

To assess the robustness of the pooled effect size, we conducted a leave-one-out sensitivity analysis (see Additional file 7). The results demonstrated that no single study had a disproportionate influence on the direction of the pooled result, as the effect sizes remained relatively stable across iterations for IFNα, IFNγ, and IL-6. However, for IL-1β, IL-12, and IL-17, the exclusion of Farid et al. [49]—a study with a notably larger sample size than others—resulted in a significant change in the effect size, indicating its significant influence. Similarly, for IL-10, the exclusion of Farid et al. [49] and Gomez et al. [61] significantly affected the effect size. In case of TNFα, the exclusion of Arora et al. [42] significantly affected the effect size.

Funnel plot asymmetry was assessed using Egger’s regression test followed by the trim-and-fill method (see Additional file 7). For IFN-γ and IL-6, Egger’s test did not indicate significant asymmetry (*p* = 0.993 and *p* = 0.717), but the trim-and-fill method suggested moderate and mild publication bias, respectively. IFN-α showed no imputed studies and no significant asymmetry (*p* = 0.344), suggesting a low risk of publication bias. Even though minimal asymmetry was detected for TNF-α (*p* = 0.240), the trim-and-fill method still imputed studies, while IL-1β and IL-17 showed significant funnel plot asymmetry in Egger’s test (*p* < 0.001 and *p* < 0.001) but only one study each was imputed for these by the trim-and-fill method. IL-10 also showed significant asymmetry (*p* = 0.030) and showed two imputed studies, whereas IL-12 although with non-significant results in Egger’s test (*p* = 0.274), had similar imputation. Overall, while some cytokines demonstrated signs of publication bias, particularly IL-1β, IL-17, and IL-10, most showed no strong evidence based on statistical tests and visual inspection.

## Discussion

In the present systematic review and meta-analysis, we quantitatively synthesized the data from 33 eligible studies, comparing IFN levels among 2307 SLE patients and 1599 HCs. The results demonstrated a significantly elevated IFNα and IFNγ levels in SLE patients compared to HCs. Further, IFNγ—but not IFNα—levels were correlated with disease activity, suggesting its potential utility as a biomarker for assessing disease activity. Additionally, we observed that IL-6 and TNFα levels were significantly elevated in SLE patients, while IL-1β, IL-10, IL-12, and IL-17 showed no statistically significant differences. Subgroup analyses revealed that differences in detection method, sample type, and geographic regions could influence measured cytokine levels.

Type I IFNs play crucial roles in regulating the immune system [14]. Their significance in SLE has been the subject of extensive investigation, with implications for assessing disease activity. Our pooled estimate revealed that type I IFN levels, i.e., IFNα, were significantly elevated in SLE cases compared with HCs. These findings align with a previous meta-analysis by Pattanaik et al., in which SLE patients presented significantly elevated levels of serum IFNα and IL-6 compared with HCs [67]. However, this study included only four studies involving 380 SLE cases and 207 HCs. Moreover, it is noteworthy that our study focused exclusively on adult SLE cases, whereas the study by Pattanaik et al. included a study involving childhood-onset SLE cases. Therefore, a more extensive meta-analysis encompassing a larger number of studies and patients was warranted to provide a comprehensive comparison of IFNα levels between SLE cases and HCs among diverse patient populations. In addition to type I IFNs, an important role of type II IFNs, specifically IFNγ, has been reported in SLE pathogenesis [11]. The present meta-analysis revealed significantly elevated IFNγ levels in SLE cases compared with HCs. Notably, these elevated levels were found to be associated with disease activity. In addition to type I and type II IFNs, few studies have reported increased levels of type III IFNs in patients with SLE [50, 52, 63]. However, due to only three studies, a meta-analysis could not be performed. The significantly elevated levels of IFNα and IFNγ in SLE cases suggest their involvement in immune dysregulation and pathogenesis of the disease. The association of IFNγ with disease activity suggests its potential as a biomarker for assessing disease activity.

The immune system, both innate and adaptive, is dysregulated in SLE. The innate immune system is activated because of the improper clearance of apoptotic debris. The self-nucleic acids released from this apoptotic debris activate plasmacytoid dendritic cells (pDCs), which are the primary producers of type I IFNs, particularly IFNα [15, 68]. Additionally, adaptive immune cells are involved in SLE. Due to the dysregulation of B- and T-cell activation in SLE, immune regulation is disrupted, resulting in the abnormal production of proinflammatory cytokines [69]. In the present meta-analysis, significantly elevated levels of IL-6 and TNFα were observed in SLE cases compared with HCs. These findings are in line with previous meta-analyses reporting elevated IL-6 levels in SLE patients [67, 70]. Other proinflammatory cytokines, such as IL-1β, IL-10, IL-12, and IL-17 have also been implicated in SLE pathogenesis. A meta-analysis by Yin et al. reported elevated IL-17 levels in patients with active SLE compared to those with inactive disease [71]. In contrast, our study did not observe a significant elevation of IL-17 or other cytokines. This discrepancy may be due to our inclusion criteria, which included studies that assessed these cytokines alongside IFNs. As a result, studies evaluating IL-17 or other cytokines in isolation or alongside cytokines other than IFNs might have been missed/excluded. Consequently, the non-significant elevation of these cytokines in the present meta-analysis warrants further investigation. The elevated levels of IL-6 and TNFα in SLE contribute to the activation of autoreactive B-cells, promoting autoantibody production and the initiation of inflammatory responses [72]. These cytokine-mediated mechanisms underscore the complexity of SLE pathogenesis and represent potential targets for therapeutic interventions.

To explore potential sources of heterogeneity in the overall pooled results, we performed subgroup analyses based on the detection method. The results demonstrated that IL-6 levels were significantly elevated irrespective of the detection method used, with reduced heterogeneity observed when ELISA was used. For IFNγ and TNFα, significant differences were observed only when ELISA was used, while results from bead-based assays were not statistically significant. These inconsistencies may be attributed to the small number of studies using bead-based assays, as well as inherent differences in assay sensitivity, specificity, and detection [73]. These results emphasize the need for more studies utilizing similar and validated assays for cytokine measurement to enhance the comparability and reproducibility across different studies.

To further explore potential sources of heterogeneity in the overall pooled results, we performed subgroup analyses based on the sample type used for the assessment of cytokines. The levels of IFNγ and TNFα were significantly elevated when serum was used, but not when plasma was used. Importantly, heterogeneity was minimal in plasma-based studies, suggesting that sample type may partly account for inter-study variability. However, the limited number of plasma-based studies restricts definitive conclusions and highlights the need for additional research using both sample types.

To evaluate the impact of regional variation on heterogeneity, subgroup analyses were performed for IFNα and IFNγ. The levels of IFNα and IFNγ were significantly elevated in the American and MENA populations, which aligns with the pooled effect size. Interestingly, heterogeneity in IFNα levels was negligible in studies from American and Asian cohorts, indicating that regional variation may partly explain the observed heterogeneity. Conversely, high heterogeneity persisted for IFNγ across all regions, suggesting the influence of additional factors such as genetic predispositions, environmental exposures, or population-specific disease characteristics [74].

Across all cytokines evaluated in the case versus control comparison, most cytokines, including IFNα, IFNγ, IL-1β, IL-10, IL-12, IL-17, and TNFα, exhibited high heterogeneity (*I*^2^ > 90%), indicating considerable variability in effect sizes across studies that could not be fully accounted for by random error. Subgroup analyses revealed that heterogeneity in cytokine levels was partially explained by detection method, sample type, and geographic region. For IL-6 and TNFα, the use of detection method and the sample type markedly influenced the heterogeneity, with *I*^2^ values dropping to 0% in some subgroups. In contrast, for IFNγ, subgrouping by these factors did not substantially reduce heterogeneity, suggesting the influence of additional, unmeasured variables. Notably, for IFNα, regional variation explained some heterogeneity, with *I*^2^ dropping to 0% in studies from America and Asia. To address the limitations of frequentist models under the conditions of high heterogeneity, we employed Bayesian hierarchical models for IFN-α, IFN-γ, IL-6, and TNF-α. These models allowed us to retain study-level variability while improving inference under uncertainty. The posterior mean estimates were generally consistent with classical random-effects estimates, with credible intervals excluding zero for all cytokines except TNF-α, reflecting greater uncertainty for that cytokine. The Bayesian framework enhanced interpretability by providing full posterior distributions for key parameters and offering a probabilistic measure of uncertainty. This approach complements traditional methods and supports the robustness of our findings for most cytokines, while highlighting greater caution for TNF-α.

Leave-one-out sensitivity analyses demonstrated the robustness of pooled estimates for IFNα, IFNγ, and IL-6, as no single study disproportionately influenced the results. However, the effect size for IL-1β, IL-10, IL-12, IL-17, and TNFα were significantly influenced by exclusion of one or more studies, particularly those with larger sample sizes or extreme effect sizes. This suggests that the conclusions related to these cytokines should be interpreted with caution.

The meta-analysis revealed elevated levels of IFNs and a few key pro-inflammatory cytokines across diverse cytokine networks in patients with SLE, suggesting their potential as therapeutic targets. Currently, many cytokine-targeting therapies are in clinical trials that have shown promising results in the management of SLE patients. Key cytokines such as IFNs, certain ILs, and B lymphocyte stimulator (BLyS) are potential targets [23, 75, 76]. For example, the recent approval of anifrolumab, a type I IFN-blocking antibody, for the treatment of SLE underscores the importance of directing therapeutic interventions toward this pathway [23, 68]. Similarly, belimumab, which targets BlysS, was the first biological agent approved for SLE, demonstrating effectiveness in lowering flare rates [75]. The effectiveness of TNF-α-targeting therapy in lupus is a subject of debate due to its both immunoregulatory and pro-inflammatory effects [2]. Infliximab and etanercept have been the most investigated anti-TNF-α drugs for lupus; however, more research is required to determine optimal treatments [77]. These results highlight the potential of cytokine-targeting treatments for the management of SLE. However, more investigations are needed to identify patient cohorts suitable for such targeted treatment.

It is important to take into account the limitations of our study while evaluating the findings. First, a notable degree of heterogeneity (*I*^2^ > 90%) was observed for most analyses. Although subgroup analyses based on detection methods, sample types, and geographic regions partially explained this variability for certain cytokines (e.g., IFNα, IL-6, and TNFα), considerable residual heterogeneity persisted, particularly for IFNγ, suggesting the influence of unmeasured confounders. Additionally, some subgroup analyses included small sample sizes, which may limit the reliability and generalizability of the pooled results. Further, the Bayesian meta-analyses complemented the traditional methods and supported the robustness of our findings for most cytokines, though results for TNFα should be interpreted with greater caution due to wider uncertainty. Second, the sensitivity analysis for IL-1β, IL-10, IL-12, IL-17, and TNFα showed that exclusion of one or more studies significantly affected the effect size, and therefore conclusions should be interpreted with caution. Third, notable publication bias was evident in the funnel plots. Egger’s regression test and the trim-and-fill method indicated that while some cytokines—particularly IL-1β, IL-17, and IL-10—exhibited signs of publication bias, most cytokines showed either no significant asymmetry or only minimal imputation. Fourth, all studies did not mention the status of multiple influencing factors, such as disease activity and type of treatment, when the levels of IFNs and cytokines were measured. Consequently, it was not feasible to ascertain whether cytokine measurements were conducted under standardized conditions across all studies. Fifth, in our meta-analysis of other cytokine levels, only those studies that assessed these cytokines alongside IFNs were included. Thus, some studies measuring these cytokines in isolation or with other cytokines might have been missed. This highlights the need for future research employing robust study designs and methodologies to increase the reliability of findings.

## Conclusions

In conclusion, to the best of our knowledge, this systematic review and meta-analysis represents the first comprehensive examination of the IFN levels in adult SLE cases in a large number of studies reported thus far. This study shows significantly elevated serum levels of IFNs and other key pro-inflammatory cytokines, suggesting their role in SLE pathogenesis. The correlation between elevated IFNγ levels and SLE disease activity suggesting its potential utility as a biomarker for monitoring SLE disease activity. While current treatment strategies often involve broad immunosuppression, there is increasing interest in targeting specific cytokines to manage lupus more effectively. Elevated levels of IFNs, IL-6, and TNFα identify these cytokines as promising future therapeutic targets for the management of SLE. Sub-group analyses revealed the effect of detection methods, biological sample type, and regional variations on cytokine levels, suggesting the need for standardized protocols to improve data comparability and reproducibility across studies. Future studies with robust study designs and methodologies are required to validate our findings due to several limitations in our study.

## Supplementary Information

Additional file 1. PRISMA 2020 checklist.

Additional file 2. Search terms and search strategy.

Additional file 3. Full length studies excluded with reasons for exclusion.

Additional file 4. Study characteristics of the included studies (a) to compare the levels of IFNs and other related cytokines between SLE cases and HCs; (b) for correlation of IFNα levels with disease activity; (c) for correlation of IFNγ levels with disease activity.

Additional file 5. Data quality of included studies.

Additional file 6. Summary statistics of meta-analysis.

Additional file 7. Funnel plots (after trim and fill method) and sensitivity analysis for comparison of (a) IFNα (b) IFNγ (c) IL-1β (d) IL-6 (e) IL-10 (f) IL-12 (g) IL-17 and (h) TNFα between SLE cases and HCs.

## Data Availability

All data generated or analyzed during this study are included in this published article [and its additional files].

## Abbreviations

BLyS: B lymphocyte stimulator
CI: Confidence interval
ELISA: Enzyme linked immunosorbent assay
HCs: Healthy controls
IFN: Interferon
IL: Interleukin
MENA: Middle East and North America
MeSH: Medical Subject Headings
NOS: Newcastle-Ottawa Quality Assessment Scale
PBMCs: Peripheral blood mononuclear cells
pDCs: Plasmacytoid dendritic cells
PRISMA: Preferred Reporting Items for Systematic Reviews and Meta-Analyses
SD: Standard deviation
SLE: Systemic lupus erythematosus
SLEDAI: Systemic Lupus Erythematosus Disease Activity Index
SMD: Standardized mean difference
TNFα: Tumor necrosis factor alpha
