# Supplementary material for: Unravelling the connection between interferons and systemic lupus erythematosus: a systematic review and meta-analysis
Source: BMC Med. 2025 Oct 8;23:543. doi: 10.1186/s12916-025-04318-1 (PMC12506321; doi:10.1186/s12916-025-04318-1)
Supplement: Supplementary file 2 — Additional file 2. Search terms and search strategy. [file 12916_2025_4318_MOESM2_ESM.docx]

**Additional file 2:** Search terms and search strategy

| **PubMed 15^th^ November, 2024** | | | |
| --- | --- | --- | --- |
| **Sr. No.** | **Key words** | **Search terms** | **Results** |
| 1 | Systemic Lupus Erythematosus | “lupus erythematosus, systemic"[MeSH Terms] OR ("lupus"[All Fields] AND "Erythematosus"[All Fields] AND "systemic"[All Fields]) OR "systemic lupus erythematosus"[All Fields] OR ("systemic"[All Fields] AND "lupus"[All Fields] AND "Erythematosus"[All Fields]) OR "SLE"[All Fields] OR ("lupus vulgaris"[MeSH Terms] OR ("lupus"[All Fields] AND "vulgaris"[All Fields]) OR "lupus vulgaris"[All Fields] OR "lupus"[All Fields] OR "lupus erythematosus, systemic"[MeSH Terms] OR ("lupus"[All Fields] AND "Erythematosus"[All Fields] AND "systemic"[All Fields]) OR "systemic lupus erythematosus"[All Fields]) OR (("lupus vulgaris"[MeSH Terms] OR ("lupus"[All Fields] AND "vulgaris"[All Fields]) OR "lupus vulgaris"[All Fields] OR "lupus"[All Fields] OR "lupus erythematosus, systemic"[MeSH Terms] OR ("lupus"[All Fields] AND "Erythematosus"[All Fields] AND "systemic"[All Fields]) OR "systemic lupus erythematosus"[All Fields]) AND "Erythematosus"[All Fields]) | 112715 |
| 2 | Interferons | "interferon s"[All Fields] OR "interferone"[All Fields] OR "interferones"[All Fields] OR "interferons"[MeSH Terms] OR "interferons"[All Fields] OR "interferon"[All Fields] OR "IFNs"[All Fields] OR ("interferon alpha"[MeSH Terms] OR "interferon alpha"[All Fields] OR ("interferon"[All Fields] AND "alpha"[All Fields]) OR "interferon alpha"[All Fields]) OR ("IFN"[All Fields] AND ("alpha"[All Fields] OR "alpha s"[All Fields] OR "alphas"[All Fields])) OR ("interferon gamma"[MeSH Terms] OR "interferon gamma"[All Fields] OR ("interferon"[All Fields] AND "gamma"[All Fields]) OR "interferon gamma"[All Fields]) OR ("IFN"[All Fields] AND ("gamma rays"[MeSH Terms] OR ("gamma"[All Fields] AND "rays"[All Fields]) OR "gamma rays"[All Fields] OR "gamma"[All Fields] OR "gamma s"[All Fields] OR "gammae"[All Fields] OR "gammas"[All Fields])) OR ("interferon lambda"[MeSH Terms] OR ("interferon"[All Fields] AND "lambda"[All Fields]) OR "interferon lambda"[All Fields]) OR ("interferon lambda"[MeSH Terms] OR ("interferon"[All Fields] AND "lambda"[All Fields]) OR "interferon lambda"[All Fields] OR ("IFN"[All Fields] AND "lambda"[All Fields]) OR "ifn lambda"[All Fields]) OR ("interferon type i"[MeSH Terms] OR "interferon type i"[All Fields] OR "type i interferons"[All Fields]) OR ("interferon type i"[MeSH Terms] OR "interferon type i"[All Fields] OR "type i ifn"[All Fields]) OR ("type"[All Fields] AND "II"[All Fields] AND ("interferon s"[All Fields] OR "interferone"[All Fields] OR "interferones"[All Fields] OR "interferons"[MeSH Terms] OR "interferons"[All Fields] OR "interferon"[All Fields])) OR ("type"[All Fields] AND "II"[All Fields] AND "IFN"[All Fields]) OR ("interferon lambda"[MeSH Terms] OR ("interferon"[All Fields] AND "lambda"[All Fields]) OR "interferon lambda"[All Fields] OR ("type"[All Fields] AND "iii"[All Fields] AND "interferons"[All Fields]) OR "type iii interferons"[All Fields]) OR ("interferon lambda"[MeSH Terms] OR ("interferon"[All Fields] AND "lambda"[All Fields]) OR "interferon lambda"[All Fields] OR ("type"[All Fields] AND "iii"[All Fields] AND "IFN"[All Fields]) OR "type iii ifn"[All Fields]) | 277202 |
| 3 | 1 AND 2 | ("lupus erythematosus, systemic"[MeSH Terms] OR ("lupus"[All Fields] AND "Erythematosus"[All Fields] AND "systemic"[All Fields]) OR "systemic lupus erythematosus"[All Fields] OR ("systemic"[All Fields] AND "lupus"[All Fields] AND "Erythematosus"[All Fields]) OR "SLE"[All Fields] OR ("lupus vulgaris"[MeSH Terms] OR ("lupus"[All Fields] AND "vulgaris"[All Fields]) OR "lupus vulgaris"[All Fields] OR "lupus"[All Fields] OR "lupus erythematosus, systemic"[MeSH Terms] OR ("lupus"[All Fields] AND "Erythematosus"[All Fields] AND "systemic"[All Fields]) OR "systemic lupus erythematosus"[All Fields]) OR (("lupus vulgaris"[MeSH Terms] OR ("lupus"[All Fields] AND "vulgaris"[All Fields]) OR "lupus vulgaris"[All Fields] OR "lupus"[All Fields] OR "lupus erythematosus, systemic"[MeSH Terms] OR ("lupus"[All Fields] AND "Erythematosus"[All Fields] AND "systemic"[All Fields]) OR "systemic lupus erythematosus"[All Fields]) AND "Erythematosus"[All Fields])) AND ("interferon s"[All Fields] OR "interferone"[All Fields] OR "interferones"[All Fields] OR "interferons"[MeSH Terms] OR "interferons"[All Fields] OR "interferon"[All Fields] OR "IFNs"[All Fields] OR ("interferon alpha"[MeSH Terms] OR "interferon alpha"[All Fields] OR ("interferon"[All Fields] AND "alpha"[All Fields]) OR "interferon alpha"[All Fields]) OR ("IFN"[All Fields] AND ("alpha"[All Fields] OR "alpha s"[All Fields] OR "alphas"[All Fields])) OR ("interferon gamma"[MeSH Terms] OR "interferon gamma"[All Fields] OR ("interferon"[All Fields] AND "gamma"[All Fields]) OR "interferon gamma"[All Fields]) OR ("IFN"[All Fields] AND ("gamma rays"[MeSH Terms] OR ("gamma"[All Fields] AND "rays"[All Fields]) OR "gamma rays"[All Fields] OR "gamma"[All Fields] OR "gamma s"[All Fields] OR "gammae"[All Fields] OR "gammas"[All Fields])) OR ("interferon lambda"[MeSH Terms] OR ("interferon"[All Fields] AND "lambda"[All Fields]) OR "interferon lambda"[All Fields]) OR ("interferon lambda"[MeSH Terms] OR ("interferon"[All Fields] AND "lambda"[All Fields]) OR "interferon lambda"[All Fields] OR ("IFN"[All Fields] AND "lambda"[All Fields]) OR "ifn lambda"[All Fields]) OR ("interferon type i"[MeSH Terms] OR "interferon type i"[All Fields] OR "type i interferons"[All Fields]) OR ("interferon type i"[MeSH Terms] OR "interferon type i"[All Fields] OR "type i ifn"[All Fields]) OR ("type"[All Fields] AND "II"[All Fields] AND ("interferon s"[All Fields] OR "interferone"[All Fields] OR "interferones"[All Fields] OR "interferons"[MeSH Terms] OR "interferons"[All Fields] OR "interferon"[All Fields])) OR ("type"[All Fields] AND "II"[All Fields] AND "IFN"[All Fields]) OR ("interferon lambda"[MeSH Terms] OR ("interferon"[All Fields] AND "lambda"[All Fields]) OR "interferon lambda"[All Fields] OR ("type"[All Fields] AND "iii"[All Fields] AND "interferons"[All Fields]) OR "type iii interferons"[All Fields]) OR ("interferon lambda"[MeSH Terms] OR ("interferon"[All Fields] AND "lambda"[All Fields]) OR "interferon lambda"[All Fields] OR ("type"[All Fields] AND "iii"[All Fields] AND "IFN"[All Fields]) OR "type iii ifn"[All Fields])) | 4927 |
| **Google Scholar 15^th^ November, 2024** | | | |
| 1 | Systemic Lupus Erythematosus | "Systemic Lupus erythematosus" OR "SLE" OR "Lupus" OR "Lupus Erythematosus" | 191000 |
| 2 | Interferons | "Interferons" OR "IFNs" OR "interferon alpha" OR "IFN alpha" OR "interferon gamma" OR "IFN gamma" OR "interferon lambda" OR "IFN lambda" OR "type I interferons" OR "type I IFNs" OR "type II interferons" OR "type II IFNs" OR "type III interferons" OR "type III IFNs" | 28100 |
| 3 | 1 AND 2 | "Systemic Lupus erythematosus" OR "SLE" OR "Lupus" OR "Lupus Erythematosus" AND "Interferons" OR "IFNs" OR "interferon alpha" OR "IFN alpha" OR "interferon gamma" OR "IFN gamma" OR "interferon lambda" OR "IFN lambda" OR "type I interferons" OR "type I IFNs" OR "type II interferons" OR "type II IFNs" OR "type III interferons" OR "type III IFNs" | 361 |
| **Scopus 15^th^ November, 2024** | | | |
| 1 | Systemic Lupus Erythematosus | TITLE-ABS-KEY ( systemic AND lupus AND erythematosus ) OR TITLE-ABS-KEY ( sle ) OR TITLE-ABS-KEY ( lupus ) OR TITLE-ABS-KEY ( lupus AND erythematosus ) ) | 163009 |
| 2 | Interferons | TITLE-ABS-KEY ( interferons ) OR TITLE-ABS-KEY ( ifns ) OR TITLE-ABS-KEY ( interferon AND alpha ) OR TITLE-ABS-KEY ( interferon AND gamma ) OR TITLE-ABS-KEY ( ifngamma ) OR TITLE-ABS-KEY ( interferon AND lambda ) OR TITLE-ABS-KEY ( ifn AND lambda ) OR TITLE-ABS-KEY ( type AND i AND interferon ) OR TITLE-ABS-KEY ( type AND i AND ifn ) OR TITLE-ABS-KEY ( type AND ii AND interferon ) OR TITLE-ABS-KEY ( type AND ii AND ifn ) OR TITLE-ABS-KEY ( type AND iii AND interferon ) OR TITLE-ABS-KEY ( type AND iii AND ifn ) ) | 453670 |
| 3 | 1 AND 2 | ( ( TITLE-ABS-KEY ( systemic AND lupus AND erythematosus ) OR TITLE-ABS-KEY ( sle ) OR TITLE-ABS-KEY ( lupus ) OR TITLE-ABS-KEY ( lupus AND erythematosus ) ) AND PUBYEAR < 2025 ) AND ( ( TITLE-ABS-KEY ( interferons ) OR TITLE-ABS-KEY ( ifns ) OR TITLE-ABS-KEY ( interferon AND alpha ) OR TITLE-ABS-KEY ( interferon AND gamma ) OR TITLE-ABS-KEY ( ifngamma ) OR TITLE-ABS-KEY ( interferon AND lambda ) OR TITLE-ABS-KEY ( ifn AND lambda ) OR TITLE-ABS-KEY ( type AND i AND interferon ) OR TITLE-ABS-KEY ( type AND i AND ifn ) OR TITLE-ABS-KEY ( type AND ii AND interferon ) OR TITLE-ABS-KEY ( type AND ii AND ifn ) OR TITLE-ABS-KEY ( type AND iii AND interferon ) OR TITLE-ABS-KEY ( type AND iii AND ifn ) ) | 10182 |

MeSH: Medical Subject Headings
