# Supplementary material for: Unravelling the connection between interferons and systemic lupus erythematosus: a systematic review and meta-analysis
Source: BMC Med. 2025 Oct 8;23:543. doi: 10.1186/s12916-025-04318-1 (PMC12506321; doi:10.1186/s12916-025-04318-1)
Supplement: Supplementary file 3 — Additional file 3. Full length studies excluded with reasons for exclusion. [file 12916_2025_4318_MOESM3_ESM.docx]

**Additional file 3:** Full length studies excluded with reasons for exclusion

| **Sr. No.** | **Reference** | **Reason for exclusion** |
| --- | --- | --- |
| 1. | Ohmura K, Iwasaki T, Oka H, et alAB1062 CORRELATION BETWEEN CYTOKINES AND CLINICAL PARAMETERS IN PATIENTS WITH ACTIVE SLE -SUBGROUP ANALYSIS USING INTERFERON ALPHA, INTERLEUKIN 6 AND INTERFERON GAMMAAnnals of the Rheumatic Diseases 2024;83:1846-1847. | Conference abstract |
| 2. | Abdulridha RH, Saud AM, Alosami MH. Evaluating treatment effect on interferon-alpha in female patients with systemic lupus erythematosus: a case-control study. J Pak Med Assoc. 2024;74(10 (Supple-8)):S210-S213. <https://doi.org/10.47391/JPMA-BAGH-16-47>. | Mean IFN levels not mentioned/insufficient data |
| 3. | Duarte-Delgado NP, Segura K, Gómez O, Pulido S, Tovar-Sánchez C, Bello-Gualtero JM, Fernández-Ávila DG, Amado-Garzón SB, Romero-Sanchez C, Cacciatore S, Rodríguez C LS. Cytokine profiles and their correlation with clinical and blood parameters in rheumatoid arthritis and systemic lupus erythematosus. Sci Rep. 2024;14(1):23475. <https://doi.org/10.1038/s41598-024-72564-z>. | Mean IFN levels not mentioned/insufficient data |
| 4. | Esparza Guerrero Y, Vazquez Villegas ML, Nava Valdivia CA, Ponce Guarneros JM, Perez Guerrero EE, Gomez Ramirez EE, Ramirez Villafaña M, Contreras Haro B, Martinez Hernandez A, Cardona Muñoz EG, Nuño Arana I, Gallardo Moya SG, Celis A, Gonzalez Lopez L, Gamez Nava JI, Saldaña Cruz AM. Association of the *STAT4* Gene rs7574865 Polymorphism with IFN-γ Levels in Patients with Systemic Lupus Erythematosus. Genes (Basel). 2023;14(3):537. <https://doi.org/10.3390/genes14030537>. | Mean IFN levels not mentioned/insufficient data |
| 5. | Damiati LA, Denetiu I, Bahlas S, Damiati S, Pushparaj PN. Immunoprofiling of cytokines, chemokines, and growth factors in female patients with systemic lupus erythematosus- a pilot study. BMC Immunol. 2023;24(1):13. <https://doi.org/10.1186/s12865-023-00551-6>. | Mean IFN levels not mentioned/insufficient data |
| 6. | Huijser E, Göpfert J, Brkic Z, van Helden-Meeuwsen CG, Jansen S, Mandl T, Olsson P, Schrijver B, Schreurs MWJ, van Daele PLA, Dik WA, Versnel MA. Serum interferon-α2 measured by single-molecule array associates with systemic disease manifestations in Sjögren's syndrome. Rheumatology (Oxford). 2022;61(5):2156-2166. <https://doi.org/10.1093/rheumatology/keab688>. | Mean IFN levels not mentioned/insufficient data |
| 7. | L Haljasmägi, H Bradford, M Menon, P Peterson, M Vanker, C Wincup, V Bondet, D Duffy, D Isenberg, K Kisand, C Mauri - S12.2 Type I interferons and their autoantibodies in the context of systemic lupus erythematosus: Lupus Science & Medicine 2022;9. | Conference abstract |
| 8. | Moreno-Torres V, Castejón R, Martínez-Urbistondo M, Gutiérrez-Rojas Á, Vázquez-Comendador J, Tutor P, Durán-Del Campo P, Mellor-Pita S, Rosado S, Vargas-Núñez JA. Serum cytokines to predict systemic lupus erythematosus clinical and serological activity. Clin Transl Sci. 2022;15(7):1676-1686. <https://doi.org/10.1111/cts.13283>. | Study without healthy controls |
| 9. | Zečević L, Begić E, Aziri B, Aganović-Mušinović I. Interferon alpha and non-specific markers of inflammation in patients with systemic lupus erythematosus. Med Glas (Zenica). 2022;19(2). <https://doi.org/10.17392/1470-22>. | Mean IFN levels not mentioned/insufficient data |
| 10. | Chasset F, Mathian A, Dorgham K, Ribi C, Trendelenburg M, Huynh-Do U, Roux-Lombard P, Courvoisier DS, Amoura Z, Gorochov G, Chizzolini C. Serum interferon-α levels and IFN type I-stimulated genes score perform equally to assess systemic lupus erythematosus disease activity. Ann Rheum Dis. 2022;81(6):901-903. <https://doi.org/10.1136/annrheumdis-2021-221835>. | Mean IFN levels not mentioned/insufficient data |
| 11. | Kailashiya V, Singh U, Kailashiya J. CTLA4 Gene Polymorphism and its Association with Disease Occurrence, Clinical Manifestations, Serum Markers and Cytokine Levels in SLE Patients from North India. Indian J Dermatol. 2022;67(3):311. <https://doi.org/10.4103/ijd.ijd_82_22>. | Mean IFN levels not mentioned/insufficient data |
| 12. | Enocsson H, Wetterö J, Eloranta ML, Gullstrand B, Svanberg C, Larsson M, Bengtsson AA, Rönnblom L, Sjöwall C. Comparison of Surrogate Markers of the Type I Interferon Response and Their Ability to Mirror Disease Activity in Systemic Lupus Erythematosus. Front Immunol. 2021;12:688753. <https://doi.org/10.3389/fimmu.2021.688753>. | Mean IFN levels not mentioned/insufficient data |
| 13. | Hu FQ, Zhang YP, Yin J, Tang ZQ, Han YF, Shi ZR, Tan GZ, Wang L. Characterization of autoantibodies and cytokines related to cutaneous lupus erythematosus. Lupus. 2021;30(2):315-319. <https://doi.org/10.1177/0961203320967759>. | Mean IFN levels not mentioned/insufficient data |
| 14. | Awalia A, Notopuro H, Soeroso J. Association of Polymorphisms in C-Reactive Protein (CRP) Promoter -821 A>G, -390 C>A/T, and Plasma Interferon-α (IFN-α) with Plasma CRP Level in Javanese Systemic Lupus Erythematosus (SLE) Patients. Acta Med Indones. 2021;53(3):261-267. | Study without healthy controls |
| 15. | Paradowska-Gorycka A, Wajda A, Stypinska B, Walczuk E, Rzeszotarska E, Walczyk M, Haladyj E, Romanowska-Prochnicka K, Felis-Giemza A, Lewandowska A, Olesińska M. Variety of endosomal TLRs and Interferons (IFN-α, IFN-β, IFN-γ) expression profiles in patients with SLE, SSc and MCTD. Clin Exp Immunol. 2021;204(1):49-63. <https://doi.org/10.1111/cei.13566>. | Mean IFN levels not mentioned/insufficient data |
| 16. | Park J, Jang W, Park HS, Park KH, Kwok SK, Park SH, Oh EJ. Cytokine clusters as potential diagnostic markers of disease activity and renal involvement in systemic lupus erythematosus. J Int Med Res. 2020;48(6):300060520926882. <https://doi.org/10.1177/0300060520926882>. | Mean IFN levels not mentioned/insufficient data |
| 17. | Fayed A, El Menyawi MM, Ghanema M, Shaker O, Elgohary R. Measurement of serum interferon alpha in Egyptian patients with systemic lupus erythematosus and evaluation of its effect on disease activity: a case-control study. Reumatismo. 2020;72(3):145-153. <https://doi.org/10.4081/reumatismo.2020.1308>. | Mean IFN levels not mentioned/insufficient data |
| 18. | Al-Hasso IK, Al-Derzi AR, Abbas AA, Gorial FI, Alnuimi AS. The role of microRNAs (MiR-125a and MiR-146a), RANTES, and IFN-γ in systemic lupus erythematosus. Ann Trop Med Public Health. 2020;23(13):231-382. <https://doi.org/10.36295/ASRO.2020.231382>. | Mean IFN levels not mentioned/insufficient data |
| 19. | ElAggan H, Farahat N, Sakr M, Tawfik S. Peripheral Blood Toll Like Receptor 7 Expression and Serum Interferon Lambda 1 Levels in Systemic Lupus Erythematosus and Their Relation to Disease Activity and Lupus Nephritis [abstract]. *Arthritis Rheumatol.* 2019; 71 (suppl 10). | Conference abstract |
| 20. | Oon S, Monaghan K, Ng M, Hoi A, Morand E, Vairo G, Maraskovsky E, Nash AD, Wicks IP, Wilson NJ. A potential association between IL-3 and type I and III interferons in systemic lupus erythematosus. Clin Transl Immunology. 2019;8(12):e01097. <https://doi.org/10.1002/cti2.1097>. | Mean IFN levels not mentioned/insufficient data |
| 21. | Mathian A, Mouries-Martin S, Dorgham K, Devilliers H, Yssel H, Garrido Castillo L, Cohen-Aubart F, Haroche J, Hié M, Pineton de Chambrun M, Miyara M, Pha M, Rozenberg F, Gorochov G, Amoura Z. Ultrasensitive serum interferon-α quantification during SLE remission identifies patients at risk for relapse. Ann Rheum Dis. 2019;78(12):1669-1676. <https://doi.org/10.1136/annrheumdis-2019-215571>. | Mean IFN levels not mentioned/insufficient data |
| 22. | Thanarajasingam U, Muppirala AN, Jensen MA, Ghodke-Puranik Y, Dorschner JM, Vsetecka DM, Amin S, Makol A, Ernste F, Osborn T, Moder K, Chowdhary V, Niewold TB. Type I Interferon Predicts an Alternate Immune System Phenotype in Systemic Lupus Erythematosus. ACR Open Rheumatol. 2019;1(8):499-506. <https://doi.org/10.1002/acr2.11073>. | Mean IFN levels not mentioned/insufficient data |
| 23. | Adhya Z, El Anbari M, Anwar S, Mortimer A, Marr N, Karim MY. Soluble TNF-R1, VEGF and other cytokines as markers of disease activity in systemic lupus erythematosus and lupus nephritis. Lupus. 2019;28(6):713-721. <https://doi.org/10.1177/0961203319845487>. | Study without healthy controls |
| 24. | Uzrail AH, Assaf AM, Abdalla SS. Correlations of Expression Levels of a Panel of Genes (*IRF5*, *STAT4*, *TNFSF4*, *MECP2*, and *TLR7*) and Cytokine Levels (IL-2, IL-6, IL-10, IL-12, IFN-*γ*, and TNF-*α*) with Systemic Lupus Erythematosus Outcomes in Jordanian Patients. Biomed Res Int. 2019;2019:1703842. <https://doi.org/10.1155/2019/1703842>. | Mean IFN levels not mentioned/insufficient data |
| 25. | Ruchakorn N, Ngamjanyaporn P, Suangtamai T, Kafaksom T, Polpanumas C, Petpisit V, Pisitkun T, Pisitkun P. Performance of cytokine models in predicting SLE activity. Arthritis Res Ther. 2019;21(1):287. <https://doi.org/10.1186/s13075-019-2029-1>. | Mean IFN levels not mentioned/insufficient data |
| 26. | Raymond WD, Eilertsen GØ, Nossent J. Principal component analysis reveals disconnect between regulatory cytokines and disease activity in Systemic Lupus Erythematosus. Cytokine. 2019;114:67-73. <https://doi.org/10.1016/j.cyto.2018.10.013>. | Mean IFN levels not mentioned/insufficient data |
| 27. | Mathian A, Mouries-Martin S, Dorgham K, Devilliers H, Barnabei L, Ben Salah E, Cohen-Aubart F, Garrido Castillo L, Haroche J, Hie M, Pineton de Chambrun M, Miyara M, Sterlin D, Pha M, Lê Thi Huong D, Rieux-Laucat F, Rozenberg F, Gorochov G, Amoura Z. Monitoring Disease Activity in Systemic Lupus Erythematosus With Single-Molecule Array Digital Enzyme-Linked Immunosorbent Assay Quantification of Serum Interferon-α. Arthritis Rheumatol. 2019;71(5):756-765. <https://doi.org/10.1002/art.40792>. | Mean IFN levels not mentioned/insufficient data |
| 28. | Torell F, Eketjäll S, Idborg H, Jakobsson PJ, Gunnarsson I, Svenungsson E, Trygg J. Cytokine Profiles in Autoantibody Defined Subgroups of Systemic Lupus Erythematosus. J Proteome Res. 2019;18(3):1208-1217. <https://doi.org/10.1021/acs.jproteome.8b00811>. | Mean IFN levels not mentioned/insufficient data |
| 29. | Tanaka A, Ito T, Kibata K, Inagaki-Katashiba N, Amuro H, Nishizawa T, Son Y, Ozaki Y, Nomura S. Serum high-mobility group box 1 is correlated with interferon-α and may predict disease activity in patients with systemic lupus erythematosus. Lupus. 2019;28(9):1120-1127. <https://doi.org/10.1177/0961203319862865>. | Reported zero as mean IFN levels in HCs |
| 30. | Fernández Matilla M, Grau García E, Fernández-Llanio Comella N, Chalmeta Verdejo I, Ivorra Cortés J, Castellano Cuesta JA, Román Ivorra JA. Increased interferon-1α, interleukin-10 and BLyS concentrations as clinical activity biomarkers in systemic lupus erythematosus. Med Clin (Barc). 2019;153(6):225-231. English, Spanish. <https://doi.org/10.1016/j.medcli.2018.12.012>. | Reported zero as mean IFN levels in HCs |
| 31. | Reynolds JA, McCarthy EM, Haque S, Ngamjanyaporn P, Sergeant JC, Lee E, Lee E, Kilfeather SA, Parker B, Bruce IN. Cytokine profiling in active and quiescent SLE reveals distinct patient subpopulations. Arthritis Res Ther. 2018;20(1):173. <https://doi.org/10.1186/s13075-018-1666-0>. | Mean IFN levels not mentioned/insufficient data |
| 32. | V Oke, I Gunnarsson, J Dorschner, A Zickert, TB Niewold, E Svenungsson - S5A:4 Circulating type i, ii and iii interferons (ifns) associate with ifn-scores, but define distinct subsets of active sle: Lupus Science & Medicine 2018;5:. | Conference abstract |
| 33. | Chen JY, Wang CM, Chen TD, Jan Wu YJ, Lin JC, Lu LY, Wu J. Interferon-λ3/4 genetic variants and interferon-λ3 serum levels are biomarkers of lupus nephritis and disease activity in Taiwanese. Arthritis Res Ther. 2018;20(1):193. <https://doi.org/10.1186/s13075-018-1683-z>. | Mean IFN levels not mentioned/insufficient data |
| 34. | Kokic V, Martinovic Kaliterna D, Radic M, Tandara L, Perkovic D. Association between vitamin D, oestradiol and interferon-gamma in female patients with inactive systemic lupus erythematosus: A cross-sectional study. J Int Med Res. 2018;46(3):1162-1171. <https://doi.org/10.1177/0300060517734686>. | Mean IFN levels not mentioned/insufficient data |
| 35. | Garcia EG, Ortiz-Sanjuan F, Matilla MF, et alFRI0275 Systemic lupus erythematosus patients with positives autoantibodies with remission or low activity exhibit both lower interferon alpha and interleukin-10 levelsAnnals of the Rheumatic Diseases 2017;76:590. | Conference abstract |
| 36. | Garcia EG, Matilla MF, Olmos CF, et alAB0494 Increased levels of interferon alpha and interleukin-10 as clinical activity biomarkers in systemic lupus erythematous patientsAnnals of the Rheumatic Diseases 2017;76:1224. | Conference abstract |
| 37. | Rose T, Grützkau A, Klotsche J, Enghard P, Flechsig A, Keller J, Riemekasten G, Radbruch A, Burmester GR, Dörner T, Hiepe F, Biesen R. Are interferon-related biomarkers advantageous for monitoring disease activity in systemic lupus erythematosus? A longitudinal benchmark study. Rheumatology (Oxford). 2017;56(9):1618-1626. <https://doi.org/10.1093/rheumatology/kex220>. | Study without healthy controls |
| 38. | Oke V, Brauner S, Larsson A, Gustafsson J, Zickert A, Gunnarsson I, Svenungsson E. IFN-λ1 with Th17 axis cytokines and IFN-α define different subsets in systemic lupus erythematosus (SLE). Arthritis Res Ther. 2017;19(1):139. <https://doi.org/10.1186/s13075-017-1344-7>. | Mean IFN levels not mentioned/insufficient data |
| 39. | Wirestam L, Enocsson H, Skogh T, Eloranta ML, Rönnblom L, Sjöwall C, Wetterö J. Interferon-α coincides with suppressed levels of pentraxin-3 (PTX3) in systemic lupus erythematosus and regulates leucocyte PTX3 in vitro. Clin Exp Immunol. 2017;189(1):83-91. <https://doi.org/10.1111/cei.12957>. | Mean IFN levels not mentioned/insufficient data |
| 40. | Amezcua-Guerra LM, Márquez-Velasco R, Chávez-Rueda AK, Castillo-Martínez D, Massó F, Páez A, Colín-Fuentes J, Bojalil R. Type III Interferons in Systemic Lupus Erythematosus: Association Between Interferon λ3, Disease Activity, and Anti-Ro/SSA Antibodies. J Clin Rheumatol. 2017;23(7):368-375. <https://doi.org/10.1097/RHU.0000000000000581>. | Mean IFN levels not mentioned/insufficient data |
| 41. | Rodero MP, Decalf J, Bondet V, Hunt D, Rice GI, Werneke S, McGlasson SL, Alyanakian MA, Bader-Meunier B, Barnerias C, Bellon N, Belot A, Bodemer C, Briggs TA, Desguerre I, Frémond ML, Hully M, van den Maagdenberg AMJM, Melki I, Meyts I, Musset L, Pelzer N, Quartier P, Terwindt GM, Wardlaw J, Wiseman S, Rieux-Laucat F, Rose Y, Neven B, Hertel C, Hayday A, Albert ML, Rozenberg F, Crow YJ, Duffy D. Detection of interferon alpha protein reveals differential levels and cellular sources in disease. J Exp Med. 2017;214(5):1547-1555. <https://doi.org/10.1084/jem.20161451>. | Mean IFN levels not mentioned/insufficient data |
| 42. | Pacheco Y, Barahona-Correa J, Monsalve DM, Acosta-Ampudia Y, Rojas M, Rodríguez Y, Saavedra J, Rodríguez-Jiménez M, Mantilla RD, Ramírez-Santana C, Molano-González N, Anaya JM. Cytokine and autoantibody clusters interaction in systemic lupus erythematosus. J Transl Med. 2017;15(1):239. <https://doi.org/10.1186/s12967-017-1345-y>. | Reported zero as mean IFN levels in HCs |
| 43. | Guimarães PM, Scavuzzi BM, Stadtlober NP, Franchi Santos LFDR, Lozovoy MAB, Iriyoda TMV, Costa NT, Reiche EMV, Maes M, Dichi I, Simão ANC. Cytokines in systemic lupus erythematosus: far beyond Th1/Th2 dualism lupus: cytokine profiles. Immunol Cell Biol. 2017;95(9):824-831. <https://doi.org/10.1038/icb.2017.53>. | Reported zero as mean IFN levels in HCs |
| 44. | Thanarajasingam U, Jensen MA, Dorschner JM, Vsetecka D, Amin S, Makol A, Ernste FC, Osborn T, Chowdhary V, Niewold TB. Hyper-Responsiveness to TLR-4 Stimulation in SLE: Association with High Levels of Serum IFN-Alpha and a Distinct Inflammatory Cytokine Profile [abstract]. *Arthritis Rheumatol.* 2016; 68 (suppl 10). | Conference abstract |
| 45. | Zickert A, Oke V, Parodis I, Svenungsson E, Sundström Y, Gunnarsson I. Interferon (IFN)-λ is a potential mediator in lupus nephritis. Lupus Sci Med. 2016;3(1):e000170. <https://doi.org/10.1136/lupus-2016-000170>. | Mean IFN levels not mentioned/insufficient data |
| 46. | Sigdel KR, Duan L, Wang Y, Hu W, Wang N, Sun Q, Liu Q, Liu X, Hou X, Cheng A, Shi G, Zhang Y. Serum Cytokines Th1, Th2, and Th17 Expression Profiling in Active Lupus Nephritis-IV: From a Southern Chinese Han Population. Mediators Inflamm. 2016;2016:4927530. <https://doi.org/10.1155/2016/4927530>. | Mean IFN levels not mentioned/insufficient data |
| 47. | López P, Rodríguez-Carrio J, Caminal-Montero L, Mozo L, Suárez A. A pathogenic IFNα, BLyS and IL-17 axis in Systemic Lupus Erythematosus patients. Sci Rep. 2016;6:20651. <https://doi.org/10.1038/srep20651>. | Mean IFN levels not mentioned/insufficient data |
| 48. | Kokic V, Martinovic Kaliterna D, Radic M, Perkovic D, Cvek M, Capkun V. Relationship between vitamin D, IFN-γ, and E2 levels in systemic lupus erythematosus. Lupus. 2016;25(3):282-8. <https://doi.org/10.1177/0961203315605367>. | Mean IFN levels not mentioned/insufficient data |
| 49. | Orrego A, Ceballos A, León AL, Lujan TP, Ortiz B and Vasquez G (2015). Levels of interferon alpha (IFN α) in patients with Systemic Lupus Erythematosus and its correlation with disease activity. *Front. Immunol. Conference Abstract: IMMUNOCOLOMBIA2015 - 11th Congress of the Latin American Association of Immunology - 10o. Congreso de la Asociación Colombiana de Alergia, Asma e Inmunología.*doi: 10.3389/conf.fimmu.2015.05.00206. | Conference abstract |
| 50. | Thanarajasingam U, Jensen MA, Dorschner JM, Niewold TB. High Levels of Serum IFN-Alpha Mark a Subgroup of SLE Patients with Distinct Immunophenotypic Features and Hyperresponsiveness to Toll-like Receptor Stimulation [abstract]. *Arthritis Rheumatol.* 2015; 67 (suppl 10). | Conference abstract |
| 51. | Schneider L, Colar da Silva AC, Werres Junior LC, Alegretti AP, Pereira dos Santos AS, Santos M, Sassi R, Heemann B, Pfaffenseller B, Tavares Brenol JC, Monticielo OA. Vitamin D levels and cytokine profiles in patients with systemic lupus erythematosus. Lupus. 2015;24(11):1191-7. <https://doi.org/10.1177/0961203315584811>. | Study without healthy controls |
| 52. | Steiman AJ, Gladman DD, Ibañez D, Noamani B, Landolt-Marticorena C, Urowitz MB, Wither JE. Lack of Interferon and Proinflammatory Cyto/chemokines in Serologically Active Clinically Quiescent Systemic Lupus Erythematosus. J Rheumatol. 2015;42(12):2318-26. <https://doi.org/10.3899/jrheum.150040>. | Study without healthy controls |
| 53. | Talaat RM, Mohamed SF, Bassyouni IH, Raouf AA. Th1/Th2/Th17/Treg cytokine imbalance in systemic lupus erythematosus (SLE) patients: Correlation with disease activity. Cytokine. 2015;72(2):146-53. <https://doi.org/10.1016/j.cyto.2014.12.027>. | Mean IFN levels not mentioned/insufficient data |
| 54. | Oke, Vilija. (2014). THU0034 High Serum Levels of IFN-Lambda1/3 and IFN-Alpha Characterize Two Separate Sub-Groups among SLE Patients. Annals of the Rheumatic Diseases. 73. 188-188. 10.1136/annrheumdis-2014-eular.5823. | Conference abstract |
| 55. | Jolly M, Francis S, Aggarwal R, Mikolaitis RA, Niewold TB, Chubinskaya S, Block JA, Scanzello C, Sequeira W. Serum free light chains, interferon-alpha, and interleukins in systemic lupus erythematosus. Lupus. 2014;23(9):881-8. <https://doi.org/10.1177/0961203314530793>. | Study without healthy controls |
| 56. | Mozo L, López P, Caminal-Montero L, Rodríguez-Carrio J, Suárez A. Anti-ribosomal P antibodies are associated with elevated circulating IFNα and IL-10 levels in systemic lupus erythematosus patients. Lupus. 2014;23(14):1477-85. <https://doi.org/10.1177/0961203314546020>. | Mean IFN levels not mentioned/insufficient data |
| 57. | Lyn-Cook BD, Xie C, Oates J, Treadwell E, Word B, Hammons G, Wiley K. Increased expression of Toll-like receptors (TLRs) 7 and 9 and other cytokines in systemic lupus erythematosus (SLE) patients: ethnic differences and potential new targets for therapeutic drugs. Mol Immunol. 2014;61(1):38-43. <https://doi.org/10.1016/j.molimm.2014.05.001>. | Mean IFN levels not mentioned/insufficient data |
| 58. | Mandal M, Tripathy R, Panda AK, Pattanaik SS, Dakua S, Pradhan AK, Chakraborty S, Ravindran B, Das BK. Vitamin D levels in Indian systemic lupus erythematosus patients: association with disease activity index and interferon alpha. Arthritis Res Ther. 2014;16(1):R49. <https://doi.org/10.1186/ar4479>. | Mean IFN levels not mentioned/insufficient data |
| 59. | Enocsson H, Sjöwall C, Kastbom A, Skogh T, Eloranta ML, Rönnblom L, Wetterö J. Association of serum C-reactive protein levels with lupus disease activity in the absence of measurable interferon-α and a C-reactive protein gene variant. Arthritis Rheumatol. 2014;66(6):1568-73. <https://doi.org/10.1002/art.38408>. | Mean IFN levels not mentioned/insufficient data |
| 60. | Boghdadi G, Elewa EA. Increased serum APRIL differentially correlates with distinct cytokine profiles and disease activity in systemic lupus erythematosus patients. Rheumatol Int. 2014;34(9):1217-23. <https://doi.org/10.1007/s00296-014-3020-4>. | Mean IFN levels not mentioned/insufficient data |
| 61. | Yin Z, Huang J, He W, Cao Z, Luo X, Zhang C, Ye Z. Serum level of eight cytokines in Han Chinese patients with systemic lupus erythematosus using multiplex fluorescent microsphere method. Cent Eur J Immunol. 2014;39(2):228-35. <https://doi.org/10.5114/ceji.2014.43728>. | Mean IFN levels not mentioned/insufficient data |
| 62. | Barakat A, Eltoraby E, Shahin D, Abdelsalam A, Abdelsalam M, Shaat N, Alsamanoudy A. Role of Ghrelin Hormone in Systemic Lupus Erythematosus: Relation to Interferon alpha and Disease Activity Biomarkers. Egyptian Journal of Rheumatology and Clinical Immunology. 2014;2(1):89-96. <https://doi.org/10.21608/ejrci.2014.10428>. | Mean IFN levels not mentioned/insufficient data |
| 63. | Elewa EA, Zakaria O, Mohamed EI, Boghdadi G. The role of interleukins 4, 17 and interferon gamma as biomarkers in patients with Systemic Lupus Erythematosus and their correlation with disease activity. The Egyptian Rheumatologist. 2014;36(1):21-7. <https://doi.org/10.1016/j.ejr.2013.10.003>. | Mean IFN levels not mentioned/insufficient data |
| 64. | Rose T, Grützkau A, Hirseland H, Huscher D, Dähnrich C, Dzionek A, Ozimkowski T, Schlumberger W, Enghard P, Radbruch A, Riemekasten G, Burmester GR, Hiepe F, Biesen R. IFNα and its response proteins, IP-10 and SIGLEC-1, are biomarkers of disease activity in systemic lupus erythematosus. Ann Rheum Dis. 2013;72(10):1639-45. <https://doi.org/10.1136/annrheumdis-2012-201586>. | Mean IFN levels not mentioned/insufficient data |
| 65. | Song LJ, Liu WW, Fan YC, Qiu F, Chen QL, Li XF, Ding F. The positive correlations of apolipoprotein E with disease activity and related cytokines in systemic lupus erythematosus. Diagn Pathol. 2013;8:175. <https://doi.org/10.1186/1746-1596-8-175>. | Mean IFN levels not mentioned/insufficient data |
| 66. | Brugos B, Vincze Z, Sipka S, Szegedi G, Zeher M. Serum and urinary cytokine levels of SLE patients. Pharmazie. 2012;67(5):411-3. <https://doi.org/10.1691/ph.2012.1694>. | Mean IFN levels not mentioned/insufficient data |
| 67. | Becker-Merok A, Østli-Eilersten G, Lester S, Nossent J. Circulating interferon-α2 levels are increased in the majority of patients with systemic lupus erythematosus and are associated with disease activity and multiple cytokine activation. Lupus. 2013;22(2):155-63. <https://doi.org/10.1177/0961203312468964>. | Mean IFN levels not mentioned/insufficient data |
| 68. | Koenig KF, Groeschl I, Pesickova SS, Tesar V, Eisenberger U, Trendelenburg M. Serum cytokine profile in patients with active lupus nephritis. Cytokine. 2012;60(2):410-6. <https://doi.org/10.1016/j.cyto.2012.07.004>. | Mean IFN levels not mentioned/insufficient data |
| 69. | Willis R, Seif AM, McGwin G Jr, Martinez-Martinez LA, González EB, Dang N, Papalardo E, Liu J, Vilá LM, Reveille JD, Alarcón GS, Pierangeli SS. Effect of hydroxychloroquine treatment on pro-inflammatory cytokines and disease activity in SLE patients: data from LUMINA (LXXV), a multiethnic US cohort. Lupus. 2012;21(8):830-5. <https://doi.org/10.1177/0961203312437270>. | Mean IFN levels not mentioned/insufficient data |
| 70. | Fragoso-Loyo H, Atisha-Fregoso Y, Núñez-Alvarez CA, Llorente L, Sánchez-Guerrero J. Utility of interferon-α as a biomarker in central neuropsychiatric involvement in systemic lupus erythematosus. J Rheumatol. 2012;39(3):504-9. <https://doi.org/10.3899/jrheum.110983>. | Mean IFN levels not mentioned/insufficient data |
| 71. | Wu Q, Yang Q, Lourenco E, Sun H, Zhang Y. Interferon-lambda1 induces peripheral blood mononuclear cell-derived chemokines secretion in patients with systemic lupus erythematosus: its correlation with disease activity. Arthritis Res Ther. 2011;13(3):R88. <https://doi.org/10.1186/ar3363>. | Mean IFN levels not mentioned/insufficient data |
| 72. | Salloum R, Franek BS, Kariuki SN, Rhee L, Mikolaitis RA, Jolly M, Utset TO, Niewold TB. Genetic variation at the IRF7/PHRF1 locus is associated with autoantibody profile and serum interferon-alpha activity in lupus patients. Arthritis Rheum. 2010;62(2):553-61. <https://doi.org/10.1002/art.27182>. | Mean IFN levels not mentioned/insufficient data |
| 73. | Mok MY, Wu HJ, Lo Y, Lau CS. The relation of interleukin 17 (IL-17) and IL-23 to Th1/Th2 cytokines and disease activity in systemic lupus erythematosus. J Rheumatol. 2010;37(10):2046-52. <https://doi.org/10.3899/jrheum.100293>. | Mean IFN levels not mentioned/insufficient data |
| 74. | Sultan MM, Al Salam FM, Hassan DA, Abdulla HS, El-Rahim A, Marwa M, Mansour HH, Hussein NR. Assessment of Proinflammatory Th1 Cytokines (IL18-IFNγ) and Th2‌ Cytokine (IL13) Concentrations in patients with Autoimmune Rheumatic Diseases (Systemic Lupus Erythematosus, Rheumatoid Artharitis and Systemic Sclerosis). The Egyptian Journal of Hospital Medicine. 2010;38(1):1-2. <https://doi.org/10.21608/ejhm.2010.17362>. | Mean IFN levels not mentioned/insufficient data |
| 75. | Lood C, Amisten S, Gullstrand B, Jönsen A, Allhorn M, Truedsson L, Sturfelt G, Erlinge D, Bengtsson AA. Platelet transcriptional profile and protein expression in patients with systemic lupus erythematosus: up-regulation of the type I interferon system is strongly associated with vascular disease. Blood. 2010;116(11):1951-7. <https://doi.org/10.1182/blood-2010-03-274605>. | Reported zero as mean IFN levels in HCs |
| 76. | Yang PT, Kasai H, Zhao LJ, Xiao WG, Tanabe F, Ito M. Increased CCR4 expression on circulating CD4(+) T cells in ankylosing spondylitis, rheumatoid arthritis and systemic lupus erythematosus. Clin Exp Immunol. 2004;138(2):342-7. <https://doi.org/10.1111/j.1365-2249.2004.02617.x>. | Reported zero as mean IFN levels in HCs |
| 77. | Morimoto S, Tokano Y, Kaneko H, Nozawa K, Amano H, Hashimoto H. The increased interleukin-13 in patients with systemic lupus erythematosus: relations to other Th1-, Th2-related cytokines and clinical findings. Autoimmunity. 2001;34(1):19-25. <https://doi.org/10.3109/08916930108994122>. | Mean IFN levels not mentioned/insufficient data |
| 78. | Bengtsson AA, Sturfelt G, Truedsson L, Blomberg J, Alm G, Vallin H, Rönnblom L. Activation of type I interferon system in systemic lupus erythematosus correlates with disease activity but not with antiretroviral antibodies. Lupus. 2000;9(9):664-71. <https://doi.org/10.1191/096120300674499064>. | Study without healthy controls |
| 79. | Tokano Y, Morimoto S, Kaneko H, Amano H, Nozawa K, Takasaki Y, Hashimoto H. Levels of IL-12 in the sera of patients with systemic lupus erythematosus (SLE)--relation to Th1- and Th2-derived cytokines. Clin Exp Immunol. 1999;116(1):169-73. <https://doi.org/10.1046/j.1365-2249.1999.00862.x>. | Mean IFN levels not mentioned/insufficient data |
| 80. | Robak E, Sysa-Jedrzejewska A, Dziankowska B, Torzecka D, Chojnowski K, Robak T. Association of interferon gamma, tumor necrosis factor alpha and interleukin 6 serum levels with systemic lupus erythematosus activity. Arch Immunol Ther Exp (Warsz). 1998;46(6):375-80. | Full-text not available |
| 81. | Liu TF, Jones BM. Impaired production of IL-12 in system lupus erythematosus. II: IL-12 production in vitro is correlated negatively with serum IL-10, positively with serum IFN-gamma and negatively with disease activity in SLE. Cytokine. 1998;10(2):148-53. <https://doi.org/10.1006/cyto.1997.0269>. | Mean IFN levels not mentioned/insufficient data |
| 82. | al-Janadi M, al-Balla S, al-Dalaan A, Raziuddin S. Cytokine profile in systemic lupus erythematosus, rheumatoid arthritis, and other rheumatic diseases. J Clin Immunol. 1993;13(1):58-67. <https://doi.org/10.1007/BF00920636>. | Mean IFN levels not mentioned/insufficient data |
