# Supplementary material for: Unravelling the connection between interferons and systemic lupus erythematosus: a systematic review and meta-analysis
Source: BMC Med. 2025 Oct 8;23:543. doi: 10.1186/s12916-025-04318-1 (PMC12506321; doi:10.1186/s12916-025-04318-1)
Supplement: Supplementary file 4 — Additional file 4. Study characteristics of the included studies (a) to compare the levels of IFNs and other related cytokines between SLE cases and HCs; (b) for correlation of IFNα levels with disease activity; (c) for correlation of IFNγ levels with disease activity. [file 12916_2025_4318_MOESM4_ESM.docx]

**Additional file 4:** (a) Study characteristics of the included studies to compare the levels of IFNs and other related cytokines between SLE cases and HCs

| **Sr. No.** | **Author and Year** | **Region** | **Study Design** | **No. of SLE Cases** | **Cytokine Levels (pg/ml) in SLE Cases (Mean±SD)** | **No. of HCs** | **Cytokine Levels (pg/ml) in HCs (Mean±SD)** | **Sample Type** | **Method of Detection** | **Quality Score** |
| --- | --- | --- | --- | --- | --- | --- | --- | --- | --- | --- |
| 1. | Nasser et al, 2023 [34] | MENA | Cross-sectional | 53 | IFNα: 371.00 ± 81.00 | 20 | IFNα: 185.00 ± 19.00 | Serum | ELISA | 5 |
| 2. | Alduraibi et al, 2023 [35] | America | Cross-sectional | 62 | IFNγ: 38.81 ± 22.27;  IL-10: 10.52 ± 5.59  IL-17: 8.51 ±11.42 | 11 | IFNγ: 26.99 ± 10.36;  IL-10: 9.33 ± 7.20;  IL-17: 2.63 ±2.62 | Plasma | ELISA | 7 |
| 3. | Abdulridha et al, 2022 [36] | MENA | Case-control | 100 | IFNα: 208.70 ± 530.00 | 50 | IFNα: 63.70 ± 34.80 | Serum | ELISA | 6 |
| 4. | Mostafa et al, 2022 [37] | MENA | Cross-sectional | 60 | IFNγ: 5.85 ± 2.85 | 60 | FNγ: 0.83 ± 0.42 | Serum | Bead-based assay | 5 |
| 5. | Farid et al, 2022 [38] | MENA | Cross-sectional | 350 | IFNγ: 25.40 ± 6.40;  IL-1β: 180.20 ± 31.10;  IL-6: 39.60 ± 8.80; IL-10: 143.60 ± 16.40;  IL-12: 234.10 ± 41.60;  IL-17: 56.70 ± 16.10;  TNFα: 34.50 ± 3.10 | 250 | IFNγ: 6.40 ± 5.80;  IL-1β: 71.60 ± 22.40;  IL-6: 22.30 ± 42.90;  IL-10: 45.80 ± 44.70;  IL-12: 102.40 ± 18.70;  IL-17: 16.50 ± 9.40;  TNFα: 17.60 ± 3.60 | Serum | ELISA | 5 |
| 6. | Abdelraouf et al, 2022 [39] | MENA | Case-control | 40 | IFNλ3: 9.70 ± 12.47 | 40 | IFNλ3: 5.13 ± 1.63 | Serum | ELISA | 8 |
| 7. | Zhang et al, 2021 [40] | Asia | Case-control | 26 | IFNα: 12.63 ± 6.38 | 6 | IFNα: 7.17 ± 1.86 | Serum | ELISA | 4 |
| 8. | Simon et al, 2021 [41] | Europe | Cross-sectional | 45 | IFNγ: 26.19 ± 9.20;  IL-6: 2.60 ± 2.10;  TNFα: 9.50 ± 7.80 | 48 | IFNγ: 17.10 ± 6.90;  IL-6: 1.30 ± 0.90;  TNFα: 6.30 ± 2.80 | Serum | Bead-based assay | 4 |
| 9. | Bayoumy et al, 2021 [42] | MENA | Cross-sectional | 40 | IFNγ: 205.00 ± 65.32 | 20 | IFNγ: 57.70 ± 18.15 | Serum | ELISA | 5 |
| 10. | Jiang et al, 2021 [43] | Asia | Case-control | 97 | IFNγ: 31680.00 ± 4290.00 | 50 | IFNγ: 59820.00 ± 13390.00 | Serum | ELISA | 7 |
| 11. | Adel et al, 2020 [44] | MENA | Cohort | 82 | IFNα: 109.30 ± 32.50;  IFNλ1: 227.90 ± 144.80 | 60 | IFNα: 5.80 ± 2.10;  IFNλ1: 30.00 ± 4.10 | Serum | ELISA | 7 |
| 12. | Li et al, 2020 [45] | Asia | Cross-sectional | 29 | IFNγ: 84.95 ± 54.23;  IL-1β: 38.98 ± 24.38;  IL-6: 34.81 ± 24.12;  IL-10: 66.66 ± 38.15;  TNFα: 106.89 ± 54.32 | 18 | IFNγ: 44.40 ± 20.00;  IL-1β: 25.39 ± 8.43;  IL-6: 8.76 ± 8.33;  IL-10: 46.11 ± 18.06;  TNFα: 73.12 ± 24.59 | Plasma | Bead-based assay | 5 |
| 13. | Oke et al, 2019 [46] | Europe | Cross-sectional | 261 | IFNα: 161.40 ± 161.00;  IFNγ: 25.90 ± 79.00;  IFNλ1: 811.20 ± 1989.00 | 322 | IFNα: 45.10 ± 69.00;  IFNγ: 13.50 ± 69.00;  IFNλ1: 472.30 ± 1208.00 | Serum | IFNα and IFNλ1: ELISA  IFNγ: Bead-based assay | 6 |
| 14. | Salazar-Camarena et al, 2019 [47] | America | Cross-sectional | 36 | IFNγ: 7.09 ± 12.78;  IL-1β: 18.44 ± 262.92;  IL-6: 23.39 ± 275.76;  IL-10: 11.10± 50.10;  IL-12: 262.47 ± 1521.78;  IL-17: 10.46 ± 105.00;  TNFα: 41.95 ± 705.96 | 15 | IFNγ: 5.61 ± 2.44;  IL-1β: 0.15 ± 2.32;  IL-6: 6.20 ± 9.18;  IL-10: 5.77 ± 1.55;  IL-12: 94.62 ± 160.47;  IL-17: 3.10 ± 0.93;  TNFα: 2.98 ± 1.94 | Serum | Bead-based assay | 5 |
| 15. | Zecevic et al, 2018 [48] | Europe | Case-control | 55 | IFNα: 0.47 ± 1.63;  IL-17: 49.27 ± 45.88 | 25 | IFNα: 0.24 ± 0.44;  IL-17: 28.64 ± 21.70 | Serum | ELISA | 7 |
| 16. | Abdel Galil et al, 2018 [49] | MENA | Cross-sectional | 123 | IFNα: 57.60 ± 20.36 | 100 | IFNα: 10.63 ± 3.56 | Serum | ELISA | 5 |
| 17. | Wen et al, 2018 [50] | Asia | Cross-sectional | 50 | IFNγ: 17.92 ± 7.26 | 15 | IFNγ: 3.94 ± 1.60 | Serum | ELISA | 5 |
| 18. | Kailashiya et al, 2018 [51] | Asia | Cross-sectional | 11 | IFNγ: 20.52 ± 23.73;  IL-10: 128.06 ± 159.71 | 10 | IFNγ: 165.40 ± 465.10;  IL-10: 75.39 ± 70.00 | Serum | ELISA | 5 |
| 19. | Luo et al, 2018 [52] | Asia | Case-control | 30 | IFNγ: 68.90 ± 29.10 | 25 | IFNγ: 24.70 ± 17.60 | Plasma | ELISA | 7 |
| 20. | Postal et al, 2017 [53] | America | Cross-sectional | 20 | IFNγ: 404.91 ± 247.12;  IL-10: 62.10 ± 21.68;  IL-12: 518.16 ± 198.77;  TNFα: 434.60 ± 335.08 | 20 | IFNγ: 86.92 ± 55.53;  IL-10: 57.38 ± 18.82;  IL-12: 354.59 ± 111.43;  TNFα: 11.18 ± 8.04 | Serum | ELISA | 6 |
| 21. | Wang et al, 2015 [54] | Asia | Case-control | 50 | IFNγ: 23.64 ± 17.69;  IL-1β: 15.48 ± 4.73;  IL-6: 19.72 ± 24.00 | 19 | IFNγ: 4.88 ± 0.91;  IL-1β: 11.53 ± 0.75;  IL-6: 4.28 ± 0.92 | Serum | ELISA | 5 |
| 22. | Rasol et al, 2015 [55] | MENA | Case-control | 50 | IFNγ: 27.72 ± 5.63 | 30 | IFNγ: 21.73 ± 4.58 | Plasma | ELISA | 7 |
| 23. | Santana-de Anda et al, 2014 [56] | America | Case-control | 36 | IFNα: 160.20 ± 21.00 | 36 | IFNα: 106.10 ± 14.00 | Serum | ELISA | 7 |
| 24. | Fauchais et al, 2013 [57] | Europe | Cross-sectional | 26 | IFNγ: 136.70 ± 230.90;  IL-10: 29.40 ± 81.10 | 26 | IFNγ: 6.90 ± 10.50;  IL-10: 0.20 ± 3.30 | Serum | ELISA | 5 |
| 25. | Ma et al, 2012 [58] | Asia | Cross-sectional | 37 | IFNα: 5.48 ± 2.34;  TNFα: 16.84 ± 4.10 | 39 | IFNα: 4.64 ± 1.43;  TNFα: 14.40 ± 2.48 | Plasma | ELISA | 7 |
| 26. | Arora et al, 2012 [59] | Asia | Case-control | 40 | IFNγ: 385.62 ± 151.19;  IL-10: 196.81 ± 75.84;  TNFα: 632.72 ± 118.36 | 40 | IFNγ: 444.23 ± 121.71;  IL-10: 66.24 ± 29.75;  TNFα: 91.43 ± 30.91 | Serum | ELISA | 7 |
| 27. | Zhang et al, 2010 [60] | Asia | Case-control | 50 | IFNα: 96.60 ± 227.35;  IL-6: 14.57 ± 23.80;  IL-10: 32.74 ± 50.03 | 30 | IFNα: 5.33 ± 8.20;  IL-6: 1.64 ± 2.67;  IL-10: 3.25 ± 2.87 | Serum | ELISA | 6 |
| 28. | Zhang et al, 2009 [61] | Asia | Cohort | 50 | IFNα: 95.36 ± 223.91 | 30 | IFNα: 5.33 ± 8.20 | Serum | ELISA | 5 |
| 29. | Kwok et al, 2008 [62] | Asia | Cross-sectional | 43 | IFNα: 3.72 ± 3.89 | 26 | IFNα: 1.20 ± 3.90 | Serum | ELISA | 5 |
| 30. | Tucci et al, 2008 [63] | America | Cross-sectional | 150 | IFNγ: 509.00 ± 562.00;  IL-12: 462.00 ± 637.00 | 69 | IFNγ: 74.90 ± 61.00;  IL-12: 79.90 ± 67.00 | Serum | ELISA | 5 |
| 31. | Calvani et al, 2004 [64] | America | Cross-sectional | 133 | IFNγ: 421.00 ± 630.50 | 44 | IFNγ: 87.70 ± 87.10 | Serum | ELISA | 5 |
| 32. | Gomez et al, 2004 [65] | America | Cross-sectional | 52 | IFNγ: 31.00 ± 13.50;  IL-10: 20.40 ± 17.40;  IL-12: 380.10 ± 596.80;  TNFα: 86.60 ± 423.70 | 25 | IFNγ: 5.30 ± 8.00;  IL-10: 27.40 ± 24.00;  IL-12: 31.40 ± 27.40;  TNFα: 18.72 ± 14.30 | Serum | ELISA | 5 |
| 33. | Amerio et al, 2002 [66] | Europe | Case-control | 20 | IFNγ: 1.50 ± 1.20;  IL-1β: 4.70 ± 3.60;  IL-10: 2.80 ± 1.50;  IL-12: 83.20 ± 54.00;  TNFα: 13.30 ± 6.50 | 20 | IFNγ: 1.50 ± 0.80;  IL-1β: 4.00 ± 2.00;  IL-10: 1.60 ± 1.20;  IL-12: 23.00 ± 14.00;  TNFα: 10.00 ± 3.00 | Serum | ELISA | 7 |

1. Study characteristics of the included studies for correlation of IFNα levels with disease activity

| **Sr. No.** | **Author and Year** | **Region** | **Study Design** | **No. of cases** | **Correlation Coefficient** |
| --- | --- | --- | --- | --- | --- |
| 1. | Abdulridha et al, 2022 [36] | MENA | Case-control | 100 | 0.062 |
| 2. | Abdel Galil et al, 2018 [49] | MENA | Cross-sectional | 123 | 0.640 |
| 3. | Zhang et al, 2009 [61] | Asia | Cohort | 50 | 0.356 |

1. Study characteristics of the included studies for correlation of IFNγ levels with disease activity

| **Sr. No.** | **Author and Year** | **Country** | **Study Design** | **No. of cases** | **Correlation Coefficient** |
| --- | --- | --- | --- | --- | --- |
| 1. | Mostafa et al, 2022 [37] | MENA | Cross-sectional | 60 | 0.601 |
| 2. | Farid et al, 2022 [38] | MENA | Cross-sectional | 350 | 0.590 |
| 3. | Oke et al, 2019 [46] | Europe | Cross-sectional | 261 | 0.140 |
| 4. | Wen et al, 2018 [50] | Asia | Cross-sectional | 50 | 0.728 |
| 5. | Arora et al, 2012 [59] | Asia | Case-control | 40 | 0.583 |
