# Supplementary material for: Unravelling the connection between interferons and systemic lupus erythematosus: a systematic review and meta-analysis
Source: BMC Med. 2025 Oct 8;23:543. doi: 10.1186/s12916-025-04318-1 (PMC12506321; doi:10.1186/s12916-025-04318-1)
Supplement: Supplementary file 5 — Additional file 5. Data quality of included studies. [file 12916_2025_4318_MOESM5_ESM.docx]

**Additional file 5:** Data quality of included studies

Newcastle-Ottawa Quality Assessment Scale results for each included study

| **Sr. No.** | **Author and Year** | **Study Design** | **Selection** | **Comparability** | **Outcome** | **Total** |
| --- | --- | --- | --- | --- | --- | --- |
| 1. | Nasser et al, 2023 [34] | Cross-sectional | **★★★** | **★** | **★** | **5★** |
| 2. | Alduraibi et al, 2023 [35] | Cross-sectional | **★★★** | **★** | **★★★** | **7★** |
| 3. | Abdulridha et al, 2022 [36] | Case-control | **★★★★** |  | **★★** | **6★** |
| 4. | Mostafa et al, 2022 [37] | Cross-sectional | **★★★** | **★** | **★** | **5★** |
| 5. | Farid et al, 2022 [38] | Cross-sectional | **★★★** | **★** | **★** | **5★** |
| 6. | Abdelraouf et al, 2022 [39] | Case-control | **★★★★** | **★★** | **★★** | **8★** |
| 7. | Zhang et al, 2021 [40] | Case-control | **★** | **★** | **★★** | **4★** |
| 8. | Simon et al, 2021 [41] | Cross-sectional | **★★★** |  | **★** | **4★** |
| 9. | Bayoumy et al, 2021 [42] | Cross-sectional | **★★★** | **★** | **★** | **5★** |
| 10. | Jiang et al, 2021 [43] | Case-control | **★★★★** | **★** | **★★** | **7★** |
| 11. | Adel et al, 2020 [44] | Cohort | **★★★★** | **★** | **★★** | **7★** |
| 12. | Li et al, 2020 [45] | Cross-sectional | **★★★** | **★** | **★** | **5★** |
| 13. | Oke et al, 2019 [46] | Cross-sectional | **★★★** | **★★** | **★** | **6★** |
| 14. | Salazar-Camarena et al, 2019 [47] | Cross-sectional | **★★★** | **★** | **★** | **5★** |
| 15. | Zecevic et al, 2018 [48] | Case-control | **★★★★** | **★** | **★★** | **7★** |
| 16. | Abdel Galil et al, 2018 [49] | Cross-sectional | **★★★** | **★** | **★** | **5★** |
| 17. | Wen et al, 2018 [50] | Cross-sectional | **★★★** | **★** | **★** | **5★** |
| 18. | Kailashiya et al, 2018 [51] | Cross-sectional | **★★★** | **★** | **★** | **5★** |
| 19. | Luo et al, 2018 [52] | Case-control | **★★★★** | **★** | **★★** | **7★** |
| 20. | Postal et al, 2017 [53] | Cross-sectional | **★★★** | **★** | **★★** | **6★** |
| 21. | Wang et al, 2015 [54] | Case-control | **★★** | **★** | **★★** | **5★** |
| 22. | Rasol et al, 2015 [55] | Case-control | **★★★★** | **★** | **★★** | **7★** |
| 23. | Santana-de Anda et al, 2014 [56] | Case-control | **★★★★** | **★** | **★★** | **7★** |
| 24. | Fauchais et al, 2013 [57] | Cross-sectional | **★★★** | **★** | **★** | **5★** |
| 25. | Ma et al, 2012 [58] | Cross-sectional | **★★★** | **★** | **★★★** | **7★** |
| 26. | Arora et al, 2012 [59] | Case-control | **★★★★** | **★** | **★★** | **7★** |
| 27. | Zhang et al, 2010 [60] | Case-control | **★★★** | **★** | **★★** | **6★** |
| 28. | Zhang et al, 2009 [61] | Cohort | **★★★** | **★** | **★** | **5★** |
| 29. | Kwok et al, 2008 [62] | Cross-sectional | **★★★** | **★** | **★** | **5★** |
| 30. | Tucci et al, 2008 [63] | Cross-sectional | **★★★** | **★** | **★** | **5★** |
| 31. | Calvani et al, 2004 [64] | Cross-sectional | **★★★** | **★** | **★** | **5★** |
| 32. | Gomez et al, 2004 [65] | Cross-sectional | **★★★** | **★** | **★** | **5★** |
| 33. | Amerio et al, 2002 [66] | Case-control | **★★★★** | **★** | **★★** | **7★** |
