# Supplementary material for: Unravelling the connection between interferons and systemic lupus erythematosus: a systematic review and meta-analysis
Source: BMC Med. 2025 Oct 8;23:543. doi: 10.1186/s12916-025-04318-1 (PMC12506321; doi:10.1186/s12916-025-04318-1)
Supplement: Supplementary file 6 — Additional file 6. Summary statistics of meta-analysis. [file 12916_2025_4318_MOESM6_ESM.docx]

**Additional file 6:** Summary statistics of meta-analysis

| **Groups** | **Cytokines** | | **Studies**  **(n)** | **Cases**  **(n)** | **Controls**  **(n)** | **Test of association (Random effects model)** | | | | **Test of Heterogeneity** | | |
| --- | --- | --- | --- | --- | --- | --- | --- | --- | --- | --- | --- | --- |
|  |  |  |  |  |  | **SMD** | **95% CI** | **Z** | **p-value** | **Q statistic** | **p-value** | **I^2^ (%)** |
| **Cases VS Control** | **IFNα** | | 12 | 916 | 744 | 1.428 | [0.78, 2.08] | 4.316 | **<0.001** | 315.744 | **<0.001** | 96.516 |
|  | **IFNγ** | | 21 | 1612 | 1137 | 0.922 | [0.32, 1.52] | 3.003 | **0.003** | 836.699 | **<0.001** | 97.610 |
|  | **IL-1β** | | 5 | 485 | 322 | 1.179 | [-0.67, 3.02] | 1.253 | 0.210 | 280.931 | **<0.001** | 98.576 |
|  | **IL-6** | | 6 | 560 | 380 | 0.679 | [0.45, 0.90] | 5.914 | **<0.001** | 8.590 | 0.127 | 41.791 |
|  | **IL-10** | | 11 | 696 | 465 | 0.798 | [-0.08, 1.68] | 1.775 | 0.076 | 334.059 | **<0.001** | 97.007 |
|  | **IL-12** | | 6 | 628 | 399 | 1.325 | [-0.10, 2.75] | 1.817 | 0.069 | 330.109 | **<0.001** | 98.485 |
|  | **IL-17** | | 4 | 503 | 301 | 1.029 | [-0.61, 2.67] | 1.233 | 0.218 | 158.887 | **<0.001** | 98.112 |
|  | **TNFα** | | 9 | 629 | 475 | 1.754 | [0.25, 3.26] | 2.288 | **0.022** | 606.003 | **<0.001** | 98.680 |
| **Correlation with disease activity** | **IFNα** | | 3 | 273 | 180 | 0.400 | [-0.05, 0.85] | 1.731 | 0.083 | 43.428 | **<0.001** | 95.395 |
|  | **IFNγ** | | 5 | 761 | 687 | 0.609 | [0.30, 0.91] | 3.923 | **<0.001** | 55.827 | **<0.001** | 92.835 |
| **Sub-group analysis: Detection methods** | **IFNγ** | ELISA | 16 | 1181 | 674 | 0.912 | [0.18, 1.64] | 2.448 | **0.014** | 683.355 | **<0.001** | 97.805 |
|  |  | Bead-based assay | 5 | 431 | 463 | 0.951 | [-0.35, 2.25] | 1.435 | 0.151 | 92.226 | **<0.001** | 95.663 |
|  | **IL-6** | ELISA | 3 | 450 | 299 | 0.657 | [0.32, 0.99] | **3.824** | **<0.001** | 0.283 | 0.868 | 0.000 |
|  |  | Bead-based assay | 3 | 110 | 81 | 0.723 | [0.32, 1.13] | **3.501** | **<0.001** | 7.941 | **0.019** | 74.813 |
|  | **TNFα** | ELISA | 6 | 519 | 394 | 2.409 | [0.63, 4.19] | **2.658** | **0.008** | 469.903 | **<0.001** | 98.936 |
|  |  | Bead-based assay | 3 | 110 | 81 | 0.448 | [-2.06, 2.95] | 0.351 | 0.726 | 2.670 | 0.263 | 25.094 |
| **Sub-group analysis: Sample type** | **IFNγ** | Plasma | 4 | 171 | 84 | 1.089 | [-0.32, 2.49] | 1.514 | 0.130 | 7.751 | 0.051 | 61.295 |
|  |  | Serum | 17 | 1441 | 1053 | 0.883 | [0.20, 1.56] | **2.539** | **0.011** | 827.177 | **<0.001** | 98.066 |
|  | **TNFα** | Plasma | 2 | 66 | 57 | 0.724 | [-2.57, 4.02] | 0.431 | 0.666 | 0.001 | 0.972 | 0.000 |
|  |  | Serum | 7 | 563 | 418 | 2.051 | [0.29, 3.82] | **2.278** | **0.023** | 555.370 | **<0.001** | 98.920 |
| **Sub-group analysis: Regional variations** | **IFNα** | America | 1 | 36 | 36 | 2.999 | [0.71, 5.29] | **2.567** | **0.010** | 0.000 | 1.000 | 0.000 |
|  |  | Asia | 5 | 206 | 131 | 0.589 | [-0.42, 1.60] | 1.142 | 0.253 | 1.080 | 0.897 | 0.000 |
|  |  | Europe | 2 | 316 | 347 | 0.577 | [-0.99, 2.15] | 0.722 | 0.470 | 10.058 | **0.002** | 90.058 |
|  |  | MENA | 4 | 358 | 230 | 2.523 | [1.39, 3.65] | **4.401** | **<0.001** | 176.652 | **<0.001** | 98.302 |
|  | **IFNγ** | America | 6 | 453 | 184 | 1.007 | [0.09, 1.92] | **2.170** | **0.030** | 32.620 | **<0.001** | 84.672 |
|  |  | Asia | 7 | 307 | 177 | 0.258 | [-0.59, 1.11] | 0.597 | 0.551 | 262.756 | **<0.001** | 97.717 |
|  |  | Europe | 4 | 352 | 416 | 0.516 | [-0.59, 1.62] | 0.916 | 0.360 | 19.837 | **<0.001** | 84.877 |
|  |  | MENA | 4 | 500 | 360 | 2.333 | [1.23, 3.44] | **4.127** | **<0.001** | 51.846 | **<0.001** | 94.214 |
