# Supplementary material for: Unravelling the connection between interferons and systemic lupus erythematosus: a systematic review and meta-analysis
Source: BMC Med. 2025 Oct 8;23:543. doi: 10.1186/s12916-025-04318-1 (PMC12506321; doi:10.1186/s12916-025-04318-1)
Supplement: Supplementary file 7 — Additional file 7. Funnel plots (after trim and fill method) and sensitivity analysis for comparison of (a) IFNα (b) IFNγ (c) IL-1β (d) IL-6 (e) IL-10 (f) IL-12 (g) IL-17 and (h) TNFα between SLE cases and HCs. [file 12916_2025_4318_MOESM7_ESM.docx]

**Additional file 7:** Funnel plots (after trim and fill method) and sensitivity analysis for comparison of (a) IFNα (b) IFNγ (c) IL-1β (d) IL-6 (e) IL-10 (f) IL-12 (g) IL-17 and (h) TNFα between SLE cases and HCs.

**
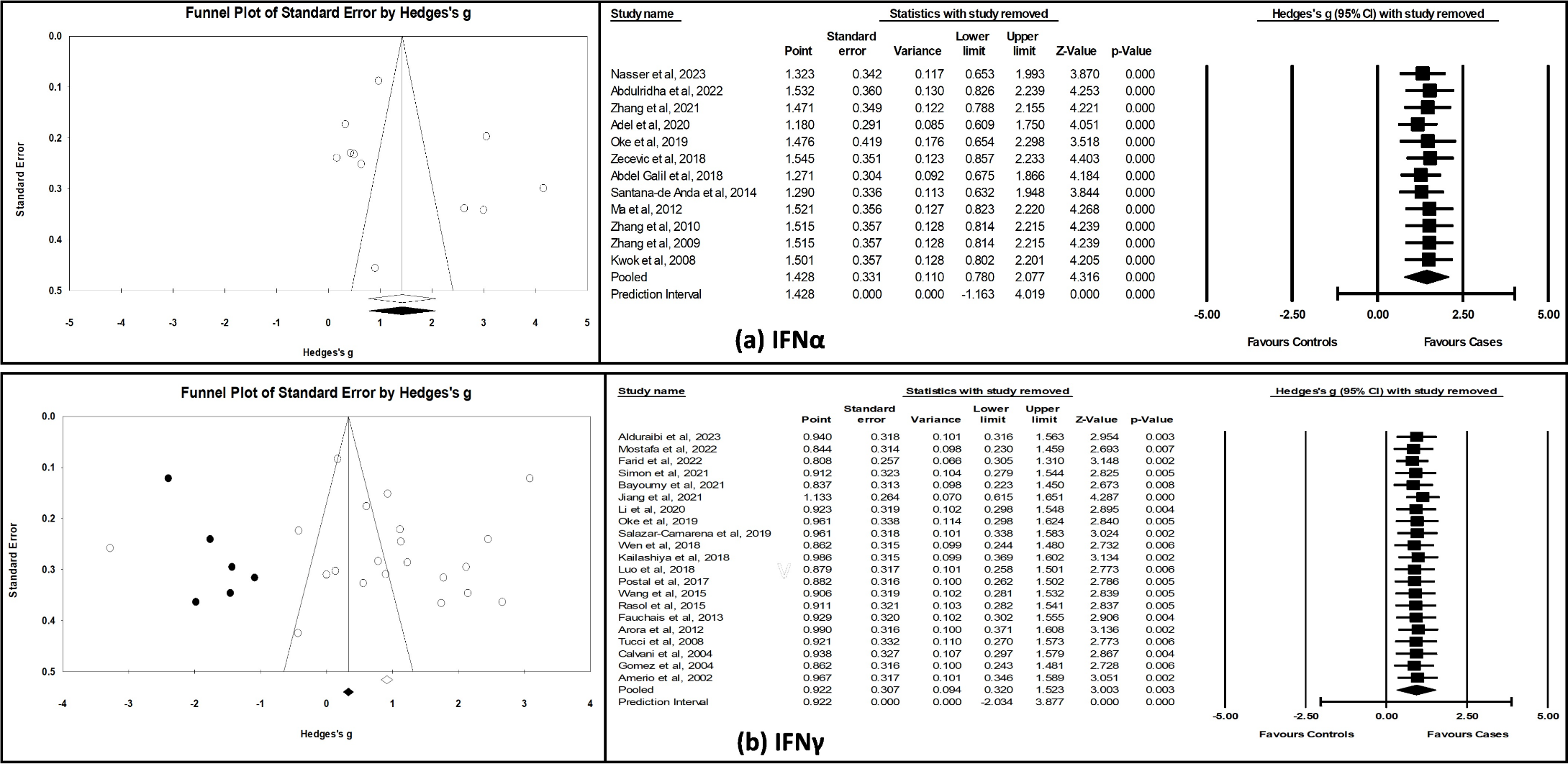
**

**
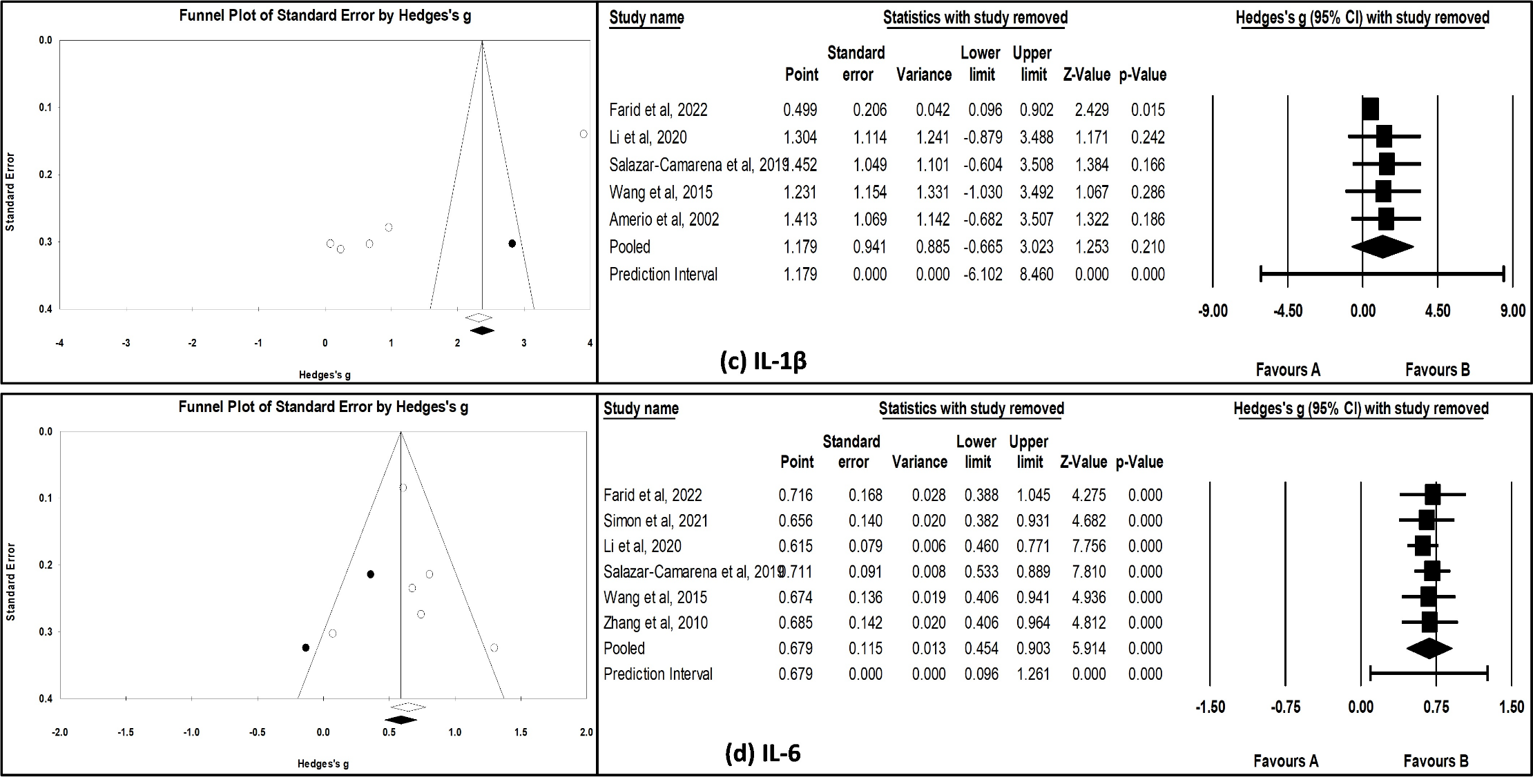
**

**
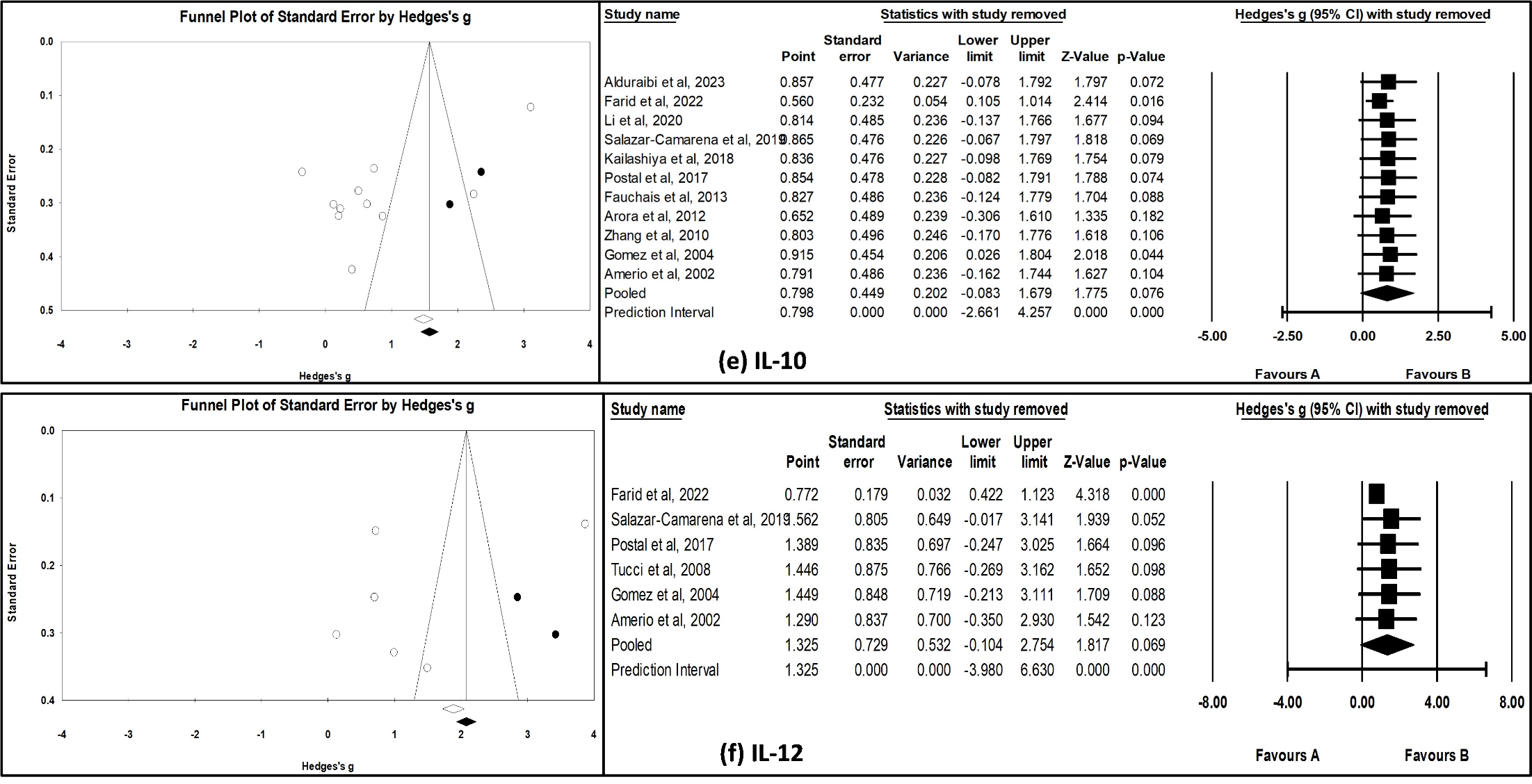
**

**
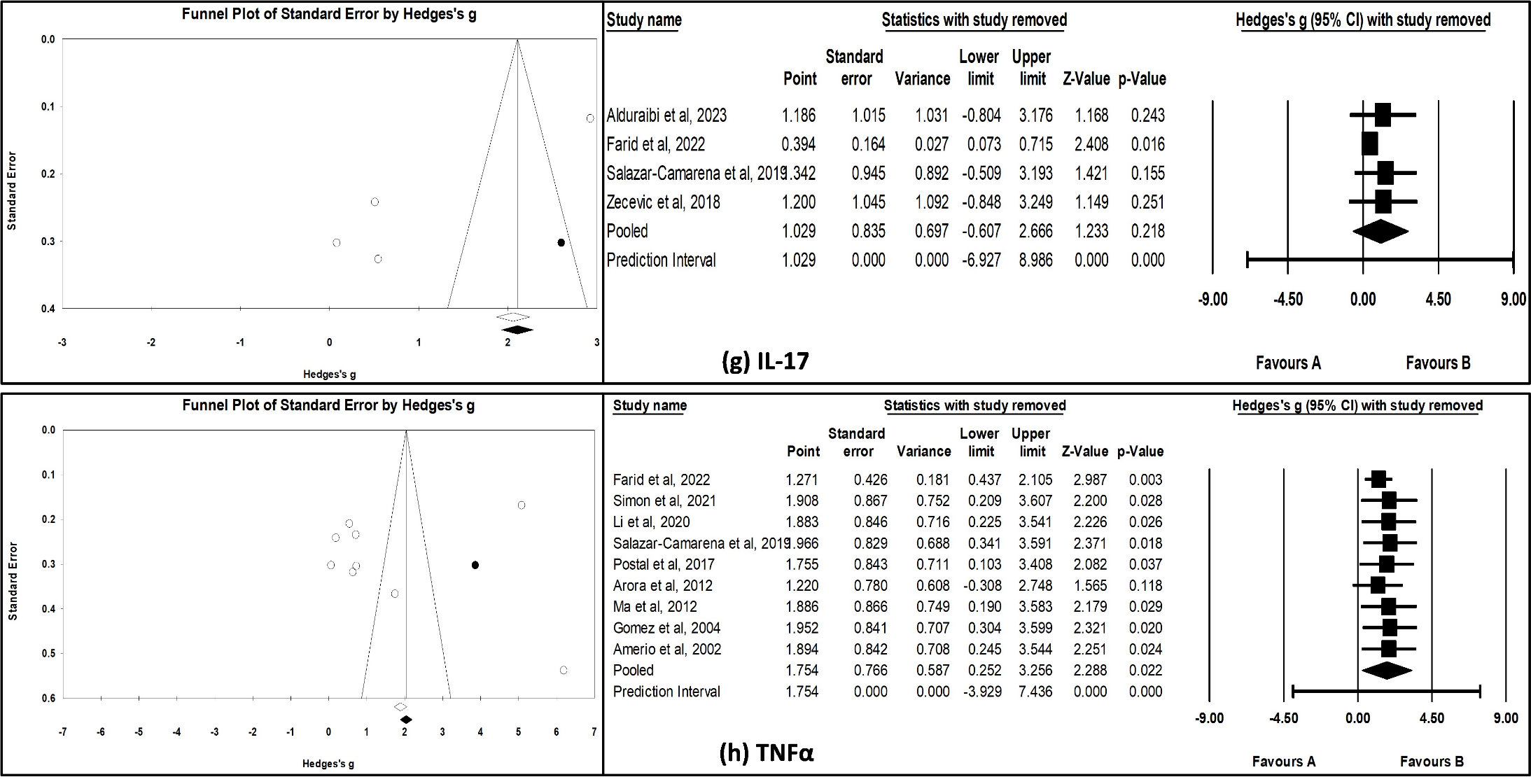
**
